# A-Kinase Anchoring Protein 2 Promotes Protection against Myocardial Infarction

**DOI:** 10.3390/cells10112861

**Published:** 2021-10-23

**Authors:** Darko Maric, Aleksandra Paterek, Marion Delaunay, Irene Pérez López, Miroslav Arambasic, Dario Diviani

**Affiliations:** 1 Department of Biomedical Sciences, Faculty of Biology and Medicine, University of Lausanne, 1011 Lausanne, Switzerland; darko.maric@unifr.ch (D.M.); aleksandra.paterek@unil.ch (A.P.); marion.delaunay@unil.ch (M.D.); irene.perezlopez@gmail.com (I.P.L.); mikiaram@gmail.com (M.A.); 2 Section of Medicine, Department of Endocrinology, Metabolism and Cardiovascular System, University of Fribourg, 1700 Fribourg, Switzerland

**Keywords:** A-kinase-anchoring protein (AKAP), protein kinase A, cAMP, cardiomyocyte, myocardial infarction, scaffolding proteins

## Abstract

Myocardial infarction (MI) is a leading cause of maladaptive cardiac remodeling and heart failure. In the damaged heart, loss of function is mainly due to cardiomyocyte death and remodeling of the cardiac tissue. The current study shows that A-kinase anchoring protein 2 (AKAP2) orchestrates cellular processes favoring cardioprotection in infarcted hearts. Induction of AKAP2 knockout (KO) in cardiomyocytes of adult mice increases infarct size and exacerbates cardiac dysfunction after MI, as visualized by increased left ventricular dilation and reduced fractional shortening and ejection fraction. In cardiomyocytes, AKAP2 forms a signaling complex with PKA and the steroid receptor co-activator 3 (Src3). Upon activation of cAMP signaling, the AKAP2/PKA/Src3 complex favors PKA-mediated phosphorylation and activation of estrogen receptor α (ERα). This results in the upregulation of ER-dependent genes involved in protection against apoptosis and angiogenesis, including Bcl2 and the vascular endothelial growth factor a (VEGFa). In line with these findings, cardiomyocyte-specific AKAP2 KO reduces Bcl2 and VEGFa expression, increases myocardial apoptosis and impairs the formation of new blood vessels in infarcted hearts. Collectively, our findings suggest that AKAP2 organizes a transcriptional complex that mediates pro-angiogenic and anti-apoptotic responses that protect infarcted hearts.

## 1. Introduction

Myocardial infarction (MI) is a leading cause of heart failure and mortality worldwide [1]. Cardiac remodeling secondary to MI promotes profound alterations in the infarcted region and areas surrounding the infarct [2]. This finally leads to infarct expansion, increased myocardial stress, ventricular dilation, fibrosis and impaired systolic and diastolic functions [3]. The major factors that influence remodeling after MI are the extent of the initial infarction and the efficiency of repair processes activated in the heart after the initial ischemic stress [3]. It has become clear that mobilization of intrinsic protective pathways in the infarcted heart can reduce cardiomyocyte apoptosis, favor angiogenesis and mitigate the negative impact of myocardial stress on cardiomyocyte function and survival [4,5]. In this context, identifying relevant cardioprotective transduction complexes activated in stressed cardiomyocytes and defining how they coordinate and transmit protective signals would provide important knowledge that could be instrumental for the development of targeted strategies of pharmacological intervention [4].

Accumulating evidence indicates that, in eukaryotic cells, the spatiotemporal control of signal transduction is ensured by scaffolding and anchoring proteins, which promote the assembly of macromolecular signaling complexes facilitating the coordination and the processing of intracellular signaling events [6,7]. A-kinase anchoring proteins (AKAPs) are molecular scaffolds that target the cyclic adenosine monophosphate (cAMP)-dependent protein kinase (PKA) and a multitude of additional signaling proteins, such as kinases, phosphatases, adenylyl cyclases, phosphodiesterases, GTPases and cytoskeletal constituents to specific cellular microdomains [8,9]. At these subcellular sites, AKAPs restrict the access of signaling molecules to selected upstream activator and downstream effectors, ensuring signal specificity and limiting the possibility of crosstalk among pathways [8,9].

AKAPs have been shown to regulate several physiological and pathophysiological functions in the heart, including action potential propagation [10,11], Ca^2+^ cycling and contractility [12,13], mitochondrial function and integrity [14,15], cardiac hypertrophy [16,17,18,19,20], cardiac fibrosis [21] and heart failure [22,23,24]. Consequently, inhibiting the expression or impairing the function of individual AKAPs can cause perturbations in heart rhythm, cardiomyocyte contraction and survival, as well as alterations in the remodeling process associated with cardiac stress [11,20,23]. In hearts subjected to stress, several AKAPs involved in the maintenance of metabolic and contractile functions of cardiomyocytes, including AKAP1 (AKAP121), AKAP5 (AKAP79) and AKAP12 (gravin), are rapidly downregulated [25,26,27,28], whereas anchoring proteins promoting cardiac remodeling and heart failure, such as AKAP6 (mAKAP), are upregulated [23]. This negatively impacts cardiomyocyte survival and eventually results in cardiac dysfunction [29,30]. 

In the present study, we investigated the AKAP2 (AKAP-KL) in MI-induced cardiac remodeling. AKAP2 was originally shown to be expressed in epithelial cells of kidneys and lungs [31], and later to play a role ocular lens transparency [32], chondrocyte function [33] and cancer-cell biology [34]. The main objectives of the present study were threefold: firstly, to determine how myocardial AKAP2 expression is regulated in response to MI; secondly, to investigate the impact of suppressing AKAP2 expression in cardiomyocytes on MI-induced myocardial damage and dysfunction; and thirdly, to determine whether anchored PKA and/or additional AKAP2-binding partners mediated the function of AKAP2 in the remodeling heart. 

## 2. Materials and Methods

### 2.1. Animal Models

LoxP-targeted *AKAP2* C57BL/6 mice were generated by Cyagen Inc. (San Diego, CA, USA). Then 5′ and 3′ homology arms, as well as a conditional knockout region (CKO) corresponding to the first exon of the AKAP2 (2.8 kb), were PCR amplified from bacterial artificial chromosome (BAC) DNA and subcloned in the targeting vector. In the final vector, the 5′ homology arm is followed by the CKO region flanked by LoxP sites, a Neomycin cassette flanked by Frt sites, and the 3′ homology arm, respectively. The targeting construct was linearized and electroporated into C57BL/6 embryonic stem (ES) cells. Neomycin resistant colonies were screened by polymerase chain reaction (PCR), using the sense primer GCTGACCGCTTCCTCGTGCTTA and the anti-sense primer CCTCATAGGAAGCGCAGATGGG. The positive clones were further analyzed by Southern Blot, using three independent probes to the verify the presence of the Neomycin cassette and to confirm proper targeting on both homologous arms. Targeted ES cell clones were injected into blastocysts, which were subsequently implanted into surrogate mothers. To delete the Neomycin cassette, chimeric males were bread to Frp-deleter C57/B6 females. The offspring was genotyped by PCR to identify germline heterozygous first generation (F1) mice carrying the floxed AKAP2 allele. The presence of the two loxP sites flanking AKAP2 exon1 was confirmed by using the following couples of primers: F1 sense primer (TTTATGCCCAATCTAGTATGCAGT) and R1 anti-sense primer (GCTAAGGAACTACCTGGTGCTCT); F2 sense primer (CCTTGCCTCTCAGTCAGCCTTG) and R2 anti-sense primer (GTATCTGCAGCCTGCAA CCCTG). Homozygous *AKAP2*^fl/fl^ mice (obtained by crossing heterozygous F1 mice) were bread with mice carrying the tamoxifen-inducible Cre-recombinase (MerCreMer) transgene driven by the alpha-myosin heavy chain promoter (Myh6-MerCreMer^+/+^) (Charles River Europe, Lyon, France) to generate double transgenic Myh6-MerCreMer^+/+^ *AKAP2*^fl/fl^ mice. The presence of the MerCreMer transgene was confirmed by PCR, using the Cre-F sense primer (ATACCGGAGATCATGCA AGC) and the Cre-R anti-sense primer (AGGTGGACCTGATCATGGAG).

### 2.2. Animal Procedures

All animal experiments were performed according to the guideline for care and use of laboratory animals and approved by the Swiss Government Veterinary Office (authorizations VD2820, VD3337 and VD3303). To achieve cardiomyocyte conditional deletion of AKAP2, 12-week-old double-transgenic Myh6-MerCreMer^+/+^ *AKAP2*^fl/fl^ mice or Myh6-MerCreMer^+/+^ control mice were injected intra-peritoneally for five consecutive days with 20 mg/kg of tamoxifen (Sigma-Aldrich, St. Louis, MO, USA). Efficiency of tamoxifen-induced exon 1 deletion was assessed by PCR performed on cardiac genomic DNA, using primers F1 and R2. 

Left anterior descending artery (LAD) ligation was performed two weeks after the last tamoxifen injection. Mice were anesthetized by intra-peritoneal injection of ketamine/xylazine/acepromazine mixture (65/15/2 mg/kg), placed on a warming pad for which temperature is maintained at 37 to 38 °C, intubated and placed on mechanical ventilation with a mini-rodent ventilator (tidal volume = 0.2 mL; rate = 120 breaths/min). The thorax of the animals was shaved and disinfected with a hydro-alcoholic solution. A left thoracotomy was performed and the fourth intercostal space was accessed by scissors and blunt dissection. The pericardium was gently opened and a pressure was applied to the right thorax to displace the heart leftward. A 7.0 silk ligature near the insertion of the left atrial appendage was placed and tied around the left descending coronary artery. Occlusion of the artery was verified by the rapid blanching of the left ventricle. Ligation was kept in place (permanent occlusion). For animals undergoing a sham operation, the ligature was placed in an identical location but not tied. The lungs were re-expanded by using positive pressure at end expiration, and the chest and skin incision were closed with 6-0 and 5-0 silk sutures, respectively. A solute of 5% glucose was administered intra- peritoneally (0.3 to 0.5 mL) after the surgery. This provides a nutritional complement to the mice during the period required for awakening. At awakening, the animals were injected subcutaneously with an analgesic drug (Temgesic, Buprenorphin, 0.1 mg/kg). This injection was repeated every 8–12 h during at least 2 days following the surgery. Two weeks after surgery mice were subjected to echocardiography as described below and sacrificed by CO_2_ inhalation and subsequent cervical dislocation. In this study a total of 153 mice were used, of which 124 survived surgery. All deaths occurred within the first 24 h after surgery. 

### 2.3. Echocardiography

Transthoracic echocardiography was performed by using a 30 MHz probe and the Vevo 2100 Ultrasound machine (VisualSonics, Toronto, ON, Canada). Mice were anesthetized with 1–1.5% isoflurane, while maintaining the heart rate at 400–500 beats per minute. The heart was imaged in 2D mode in the parasternal long-axis view. From this view, an M-mode cursor was positioned perpendicular to the interventricular septum and the posterior wall of the left ventricle at the level of the papillary muscles. Diastolic and systolic internal ventricular septum (IVS; d and IVS; s), diastolic and systolic left ventricular free posterior wall thickness (LVPW; d and LVPW; s) and left ventricular internal end-diastolic and end-systolic chamber (LVID; d and LVID; s) dimensions were measured. The measurements were taken in three separate M-mode images and averaged. Left ventricular fractional shortening (%FS) and ejection fraction (%EF) were also calculated. Fractional shortening was assessed from M-mode based on the percentage changes of left ventricular end-diastolic and end-systolic diameters. %EF was derived from the following formula: (LV vol; d–LV vol; s)/LV vol; d × 100.

### 2.4. Immunohistochemistry

Hearts were collected immediately after mouse sacrifice, washed in ice-cold phosphate buffered saline buffer (PBS) and fixed in PBS-buffered 4% formaldehyde solution. After dehydration, hearts were included in paraffin blocks. To determine cardiac AKAP2 distribution, deparaffinized transversal (short axis) 3 μm heart sections were immunostained with an affinity purified rabbit polyclonal anti-AKAP2 antibody (custom made, Covance, San Diego, CA, USA) and a horseradish peroxidase (HRP)-conjugated goat anti-rabbit secondary antibody (GE life sciences, Chicago, IL, USA, 1:500 dilution).

### 2.5. Infarct Size Determination

Hearts were collected, washed in ice-cold PBS and fixed in PBS-buffered 4% formaldehyde solution. After dehydration of the tissue, the hearts were included in paraffin blocks. For histological analysis of infarct size, transversal (short axis) 3 μm heart sections were obtained and stained with Masson Trichrome staining. Images were captured with a Zeiss Axioscan Z1 microscope. ImageJ 1.38 software was used to measure lengths of infarct and LV. The infarct size was measured by using the method described previously [35]. Briefly, the endocardial and epicardial circumferences, as well as the endocardial infarct length (scar > 50% thickness of myocardium) and epicardial infarct length (length of the transmural infarcted region) were traced manually and measured by using the ImageJ 1.38 software. Endocardial and epicardial infarct ratio were obtained by dividing the endocardial and epicardial infarct lengths by endocardial and epicardial circumferences, respectively. Finally, infarct size was calculated as the sum of endocardial and epicardial ratios, divided by two and multiplied by 100. For each heart, 3 or 4 sections were quantified and the infarct size is reported as the mean of the sections.

### 2.6. TUNEL Assay

Mice were sacrificed 24 h after LAD ligation. Hearts were collected, washed in ice-cold PBS and fixed in 4% formol PBS-buffered solution. After dehydration, the hearts were included in paraffin blocks. Terminal deoxynucleotidyl transferase dUTP nick end labeling (TUNEL) staining was performed on 1 μm transversal heart sections, using the DeadEnd^TM^ Fluorometric TUNEL System (Promega, Madison, WI, USA, n°63250) according to manufacturer’s instructions. A DAPI staining was used to visualize the nuclei. Images were captured with a Zeiss Axioscan Z1 microscope. Apoptotic nuclei were quantified using the ImageJ 1.38 software. The merge of TUNEL and DAPI stainings was first converted to 16-bits, the colors were inverted and the threshold adjusted to select only the apoptotic nuclei. The watershed tool was applied and the number of positive nuclei determined with the Analyze Particles tool. TUNEL-positive nuclei in each heart section were normalized to the number of 4’,6-diamidino-2-phénylindole (DAPI)-stained nuclei. For each heart, 5 or 6 sections cut through the infarcted area were quantified, and apoptosis was reported as the mean of the sections.

### 2.7. Lectin Staining and Vessel Density Determination

To determine vessel density, 3 µm deparaffinized transversal heart sections were incubated with biotinylated Griffonia (Bandeiraea) Simplicifolia lectin I (Vector laboratories, Burlingame, CA, USA, catalog n° B-1105) for 2 h, at room temperature, as previously described [36]. Nuclei were stained with glychemalun. HRP revelation was performed with the HRP 3,3’Diaminobenzidine (DAB+) detection system according to the manufacturer instructions. Sections were stained by the Mouse Pathology Facility (MPF) of the University of Lausanne. The number of vessels was counted for 8 to 10 random fields in the infarcted, border and remote zone of each section in both control and AKAP2 KO mice two weeks after LAD ligation. A total of 3 to 6 sections per heart were analyzed. Images were taken by using the Nikon Stereomicroscope SMZ 25 and quantified by using the ImageJ software, Plugin: Cell Counter.

### 2.8. RNA Preparation and Quantitative Real-Time PCR

The mRNAs levels of AKAP2, ANP, BNP, Myh6, Myh7, Col1a1, Col3a1, Bcl2, VEGFa, VEGFb, Cyclin D1 and Nos1 in left ventricular samples of control and cardiomyocyte-specific AKAP2 KO mice were determined by real-time RT-PCR analysis by using a Quantstudio6 real-time PCR system (Applied Biosystems, Waltham, MA, USA). Total RNA was extracted from infarcted, border or remote zones, as well as whole left ventricular tissues, using the RNeasy Fibrous Tissue Mini Kit (QIAGEN). Single-strand cDNA was synthesized from 2 μg of total RNA by using 200 ng random hexamers (Thermo Fisher Scientific, Waltham, MA, USA) and 200U SuperScript II reverse transcriptase (Thermo Fisher Scientific). Reverse transcription (RT)-PCR reactions were prepared using 100 ng of the reverse- transcribed RNA, selected TaqMan Gene Expression Assays (Thermo Fisher Scientific) and 1× TaqMan universal PCR Mastermix (Thermo Fisher scientific). Glyceraldehyde-3-phosphate (GAPDH) dehydrogenase mRNA was used as internal control.

### 2.9. Rat neonatal Ventricular Myocytes Preparation

Rat neonatal ventricular myocytes (NVMs) were prepared from 1-to-2-day-old Sprague-Dawley rats. Excised hearts were digested by three cycles of enzymatic digestion at 37 °C for 15 min, using a mixture of 0.45 mg/mL of collagenase type II (Worthington, Columbus, OH, USA) and 1mg/mL of pancreatin (Sigma), followed by centrifugation (800 rpm, 10min). The cells contained in the final pellet were suspended in maintenance medium (80% DMEM, 20% M199 medium, 1% penicillin/streptomycin solution (Thermo Fisher Scientific), and 1% 4-(2-hydroxyethyl)-1-piperazineethanesulfonic acid (HEPES) (ThermoFisher Scientific), supplemented with 10% fetal calf serum and 5% horse serum (Thermo Fisher Scientific), and seeded on T75 cell culture flasks to deplete fibroblast. After two sequential steps of 1h of differential plating, non-adherent neonatal myocytes were seeded in cell culture dishes pre-coated with 0.2% gelatin. After 24 h the medium was changed and cells were cultured in maintenance medium supplemented with 5% horse serum. Cardiomyocyte culture purity was >95% as assessed by immunocytochemistry using an anti-α-actinin monoclonal antibody.

### 2.10. AKAP2 Immunoprecipitation and Shotgun Proteomic Analysis

Endogenous AKAP2 was immunoprecipitated either from rat neonatal ventricular cardiomyocytes (NVMs) or heart ventricular lysates. Rat NVMs were grown in 100 mm^2^ plates and lysed in 1 mL of buffer A (20 mM Tris-HCl, pH 7.4, 150 mM NaCl, 1% Triton X-100, 0.1% sodium deoxycholate, 5 mg/mL aprotinin/10 mg/mL leupeptin/1 mM PMSF) whereas heart ventricular tissues were disrupted with a Polytron homogenizer (×cycles of 10 s at 10,000 RPM) in 3 mL of buffer A. Cell lysates were incubated 2 h at 4 °C on a rotating wheel. The solubilized material was centrifuged at 100,000× *g* for 30 min at 4 °C, and the protein concentration of the supernatants was determined by using a *DC*™ Protein Assay kit (Biorad Laboratories, Hercules, CA, USA). For AKAP2 immunoprecipitations from rat NVMs lysates, 2 mg of solubilized proteins were mixed with 2 µg of either mouse anti-AKAP2 antibody (BD biosciences, Franklin Lakes, NJ, USA) antibody or normal mouse immunoglobulin G (IgG) (negative control). For shotgun analysis, 40 mg of rat heart proteins were mixed with 22 µg of AKAP2 or control antibodies. Incubations were carried on for 4 h at 4 °C on a rotating wheel. All the samples were then incubated with protein-G sepharose beads for another 2 h at 4 °C on a rotating wheel. Following a brief centrifugation on a bench-top centrifuge, the pelleted beads were washed five times with buffer A containing 300 mM NaCl and twice with buffer A. Immunoprecipitates from rat NVMs were eluted from sepharose beads by boiling the samples for 3 min, at 95 °C, and subsequently separated by sodium dodecyl sulphate–poly acrylamide gel electrophoresis (SDS–PAGE) and analyzed by Western blot. For shotgun proteomic analysis, immunoprecipitates from heart lysates were separated by limited electrophoresis after which 4–6 molecular-weight regions were cut and trypsin digested. Analysis was performed by using LC–MS/MS on every fraction. The resulting collections of spectra were pooled for every sample before database search. Lists of identified proteins for each sample with their scores were subjected to statistical validation and aligned for comparison. The mass spectrometry proteomics data were deposited into the ProteomeXchange Consortium via the proteomics identification (PRIDE) partner repository with the dataset identifier PXD028514 and 10.6019/PXD028514.

### 2.11. SDS–PAGE and Western Blotting

Rat NVMs or mouse ventricular tissue lysates were denatured in SDS–PAGE sample buffer (65 mM Tris-HCl Ph 6, 8, 2% SDS, 5% glycerol, 5% 2-mercaptoethanol) by boiling samples for 3 min at 95 °C and separated on acrylamide gels and electroblotted onto nitrocellulose membranes. The blots were incubated with primary antibodies and horseradish-conjugated secondary antibodies (GE Healthcare, Amersham, UK). The following primary antibodies were used for immunoblotting: affinity purified rabbit polyclonal anti-AKAP2 (Covance-Custom made, 1 mg/mL, 1:1000 dilution) raided against a synthetic peptide derived from the mouse AKAP2 sequence (ALQENSLADFSLPQTPQTDNPSEGR), mouse monoclonal anti-AKAP2 (BD biosciences, 1:1000 dilution, catalog n° 611134), rabbit polyclonal anti-Src3 (Cell Signaling Technologies, Danvers MA, USA, 1:1000 dilution, catalog n°2126), mouse monoclonal anti-actin (Sigma, 1:1000 dilution, catalog n°A3853), rabbit polyclonal anti-phospho estrogen receptor α, (Ser305) (Bethyl Laboratories, Montgomery, TX, USA, 1:250 dilution, catalog n° A300-598A), rabbit polyclonal anti-estrogen receptor α (Abcam, 1:1000 dilution). Rabbit and mouse IgG were purchased from Santa Cruz Biotechnology (Dallas, TX, USA, catalog n°sc-2027 and sc-2025). The secondary donkey anti-rabbit and anti-mouse HRP-linked antibodies were purchased from Amersham (catalog n°NA931-1ML and NA934-1ML).

### 2.12. Plasmids and Constructs

Double-stranded hairpin (sh) oligonucleotides based upon rat AKAP2 (NCBI reference number: NM_001011974.2: 196-2808) or Src3 (NCBI reference number: NM NM_053454.1: 223-4425) mRNA sequences were cloned into the HindIII and BglII sites in a pSUPER vector. The following oligonucleotide sequences were used: rat AKAP2 shRNA-1 (sense strand) 5′-GGTAGAACCCATTGAGAAA-3′; rat AKAP2 shRNA-2 (sense strand) 5′-GTGACAGTGGAGCTTCTAA-3′; scrambled of rat AKAP2 shRNA (sense strand) 5′- GGATGCCAAGGGTATATCT-3′. To generate lentiviral transfer vectors encoding AKAP2 or scr3 shRNAs, cDNA fragments containing the H1 RNA polymerase III promoter as well as the sequences encoding shRNAs were excised using EcoRI/KpnI from the pSUPER vector and subcloned into the pSD28-GFP transfer vectors. The pSD28-GFP vector contains a GFP cassette under the control of a CMV promoter and was used to express shRNAs directed against rat AKAP2 or src3 in primary cultures of rat cardiomyocytes. Moreover, pCMVDR8.91 and pMD2.VSVG helper vectors were previously described [37]. The 3 × ERE TATA-Luc and pGL3-RARE-Luc vectors were purchased from Addgene (Watertown, MA, USA, catalog n°11354 and n°13458), GRE-Luc and ARE-Luc were a generous gift of Prof. E. Hummler (University of Lausanne).

### 2.13. Cell Culture

The 293-LTV cells were maintained in Dulbecco’s modified Eagle medium (DMEM) (Life Technologies, Carlsbad, CA, USA, catalog n°41965-039) containing 10% fetal bovine serum (Thermo Fisher Scientific, catalog n°10270-106) and 100 µg/mL of gentamicin (Thermo Fisher Scientific, catalog n°15750-045) at 37 °C and 5% CO_2_.

### 2.14. Production of Lentiviruses

Vesicular stomatitis virus G (VSV-G) pseudotyped lentiviruses were produced by cotransfecting 293-LTV cells with 20 µg of the pSD28-GFP vector containing the cDNA encoding AKAP2 or Src3 shRNAs, 15 µg of pCMVDR8.91 and 5 µg of pMD2.VSVG using the calcium phosphate method. Cell supernatants were collected 48 h later and filtered through a 0.22 μm filter unit. Lentiviruses were concentrated by ultracentrifugation at 34,000× g and resuspended in PBS. Virus titers were determined by infecting 293-T cells, using serial dilutions of the viral stocks and by scoring the number of green fluorescent protein (GFP)-positive cells at 72 h after infection. Titers determined using this method were between 2 × 10^8^ and 1.8 × 10^9^ transducing units (TU)/mL.

### 2.15. Lentiviral Infection

Rat NVMs were infected at 90% confluency, using pSD28-based lentiviruses at a multiplicity of infection (MOI) of 25 in maintenance medium containing 5% horse serum and 8 µg/mL of polybrene (Sigma-Aldrich, catalog n°107689). Forty-eight hours after infection, rat NVMs were incubated with fresh maintenance medium for an additional 24 h. Rat NVMs were subsequently starved for 24 h in serum-free maintenance medium before stimulation with 100 nM G1, 1 µM isoproterenol or 1 µM prostaglandin E2 (PGE2).

### 2.16. Luciferase Activity Assay

We infected 1 × 10^6^ NVMs seeded in 6-well dishes, using lentiviruses as indicated above. Forty-eight hours after infection cardiomyocytes were transfected using Lipofectamine 2000 with 200 ng of the CMV-Renilla plasmid and 3800 ng of the following reporter plasmids: 3 x ERE TATA-Luc (Addgene, catalog n°11354), pGL3-RARE-Luc (Addgene, catalog n°13458), GRE-Luc and ARE-Luc (generous gift of Prof. H. Hummler, Lausanne). Cells were lysed and luciferase activity was measured according to manufacturer’s instructions (Dual-Luciferase Reporter Assay System, Promega, catalog n°E1910).

### 2.17. PKA-Anchoring Inhibitory Peptides

PKA-anchoring inhibitory peptides including RIAD (LEQYANQLADQIIEATA-R_11_), scrambled RIAD (IEKELAQQYQNADAITLEK-R_11_), super-AKAP-IS (R_11_-QIEYVAKQIVDYAIHQA) and scrambled super-AKAP-IS (R_11_-QDVEIHVKAAYYQQIAI) were synthesized by GenWay Biotech (San Diego, CA, USA). Peptides were rendered cell permeable by the addition of N- or C-terminal tails of 11 arginines. Lyophilized peptides were dissolved in DMSO and stored at −80 °C, at a concentration of 5mM.

### 2.18. ELISA Assays

Extracellular medium collected from rat NVM cultures was dialyzed in a buffer containing 10 mM Tris-HCl, pH 7.4 and 50 mM NaCl for 2 h at 4 °C and subsequently concentrated 3× through microcon 10 kDa centrifugal filter units (Sigma-Aldrich). The amount of VEGFa in concentrated supernatants was determined by using an ELISA kit for Vascular Endothelial Growth Factor A (USCN Business Co., Ltd., Wuhan, China, n° SEA143Ra) according to the manufacturer’s instructions.

### 2.19. Statistical Analysis

Values are presented as mean ± standard error of the mean (SEM). Differences in means between two groups were analyzed with unpaired 2-tailed Student’s *t*-test and those among multiple groups with 1- or 2-way ANOVA, followed by Tuckey tests with Bonferroni’s correction for multiple comparisons. Values of *p* < 0.05 were considered statistically significant.

## 3. Results

### 3.1. AKAP2 Is Upregulated in Cardiomyocytes after Myocardial Infarction

Global transcriptome analysis in the border zone of infarcted mouse hearts revealed a significant upregulation of AKAP2 mRNA fourteen days post-infarction [38], raising the hypothesis that this anchoring protein could play a role in the adaptive response occurring after myocardial injury.

**Figure 1:**
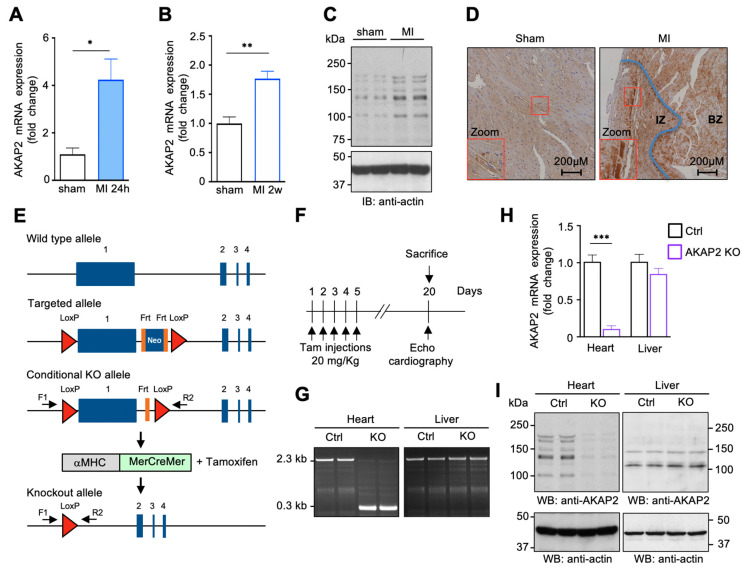
Cardiac AKAP2 is upregulated after myocardial infarction. (**A**,**B**) Quantitative real-time PCR analysis of cardiac AKAP2 mRNA expression in C57BL/6 mouse hearts subjected to sham operation or LAD ligation for 24 h (MI 24 h) (A) or 2 weeks (MI 2w) (**B**). Sham *n* = 4, MI 24 h *n* = 4, MI 2w *n* = 4. (**C**) Immunoblot analysis of AKAP2 expression in lysates from mouse hearts subjected to sham operation or LAD ligation (MI) for 2 weeks. (**D**) Anti-AKAP2 stainings of transversal heart sections from mice undergoing sham operation or LAD ligation (MI) for 2 weeks. The boundary between infarcted (IZ) and border zone (BZ) is indicated by a blue line. (**E**) Schematic representation of the AKAP2 genetic locus and the strategy used to create cardiomyocyte-specific AKAP2 knockout mice. AKAP2 exon1 is deleted following administration of tamoxifen to *AKAP2^fl/fl^*; α-MHC-MerCreMer mice. PCR primers F1 and R2 were used to assess homologous DNA recombination in cardiac tissues. (**F**) Experimental scheme for inducing AKAP2 gene deletion. (**G**) Assessing homologous DNA recombination in *AKAP2^fl/fl^*; α-MHC-MerCreMer mice. Primers F1 and R2 amplify a 2.4 kb fragment in untreated (Ctrl) mice and a 0.28 kb product following tamoxifen-induced Cre-mediated exon 1 deletion (KO). (**H**) AKAP2 mRNA expression in heart and liver lysates from control (Ctrl) and AKAP2 KO mice was assessed by quantitative real-time PCR. Ctrl *n* = 4, AKAP2 KO *n* = 4. (**I**) AKAP2 immunoblots of heart and liver lysates from control (Ctrl) and AKAP2 KO mice. Values are presented as mean ± SEM; * *p* < 0.05, ** *p* < 0.01 and *** *p* < 0.001.

To validate these findings, we subjected C57BL/6 mice to LAD ligation or to sham operation and analyzed left ventricular AKAP2 mRNA expression by qPCR at 1 day and 14 days after MI. Our results indicate that the anchoring protein was induced by 4.1 and 1.7 fold at 24 h and 14 days post-MI, respectively, suggesting that AKAP2 expression increases rapidly after myocardial injury (Figure 1A,B). Western blots performed on of heart lysates highlighted the presence of multiple AKAP2 forms, which were all upregulated following MI (Figure 1C). Interestingly, AKAP2 expression appears to increase mainly in surviving cardiomyocytes located in the infarcted zone (IZ) (Figure 1D, right panel, inset) and in the border zone (BZ) (Figure 1D, right panel). 

### 3.2. Targeted Deletion of AKAP2 in Adult Cardiomyocytes Exacerbates Cardiac Dysfunction after MI

To address the role of AKAP2 during MI, we generated a tamoxifen-inducible cardiomyocyte-specific AKAP2 knockout (KO) model in C57BL/6 mice by introducing two LoxP sites flanking AKAP2 exon 1, which represents 88% of the AKAP2 coding sequence (Figure 1E). Mice homozygous for the floxed AKAP2 allele (*AKAP2^fl/fl^*) were crossed with α-myosin heavy chain-MerCreMer transgenic mice, which express the tamoxifen-inducible Cre recombinase under the control of the cardiomyocyte-specific α-MerCreMer promoter (Figure 1E). At 8 weeks of age, *AKAP2^fl/fl^ α*MHC-MerCreMer mice were subjected to intraperitoneal tamoxifen injections as indicated (Figure 1F), resulting in cardiomyocyte-specific AKAP2 deletion (AKAP2 KO). Tamoxifen treated α-MHC-MerCreMer mice were used as controls (Ctrl). Fifteen days after the last tamoxifen injection, exon 1 deletion in ventricular tissues was confirmed by genomic PCR (Figure 1G), whereas knockout of AKAP2 mRNA and protein was assessed by qPCR (Figure 1H) and by Western blot (Figure 1I), respectively. Reduction of AKAP2 expression in ventricular lysates reached 70–80%. The residual cardiac expression could be attributed to the presence of AKAP2 in non-myocyte cells.

To evaluate the impact of suppressing AKAP2 expression in cardiomyocytes on basal heart function, transthoracic m-mode echocardiography was performed on control and AKAP2 KO mice 15 days after tamoxifen administration. Results indicate that AKAP2 deletion does not promote any significant baseline alteration of cardiac morphology and function in both male and female mice (Table 1).

**Figure 2:**
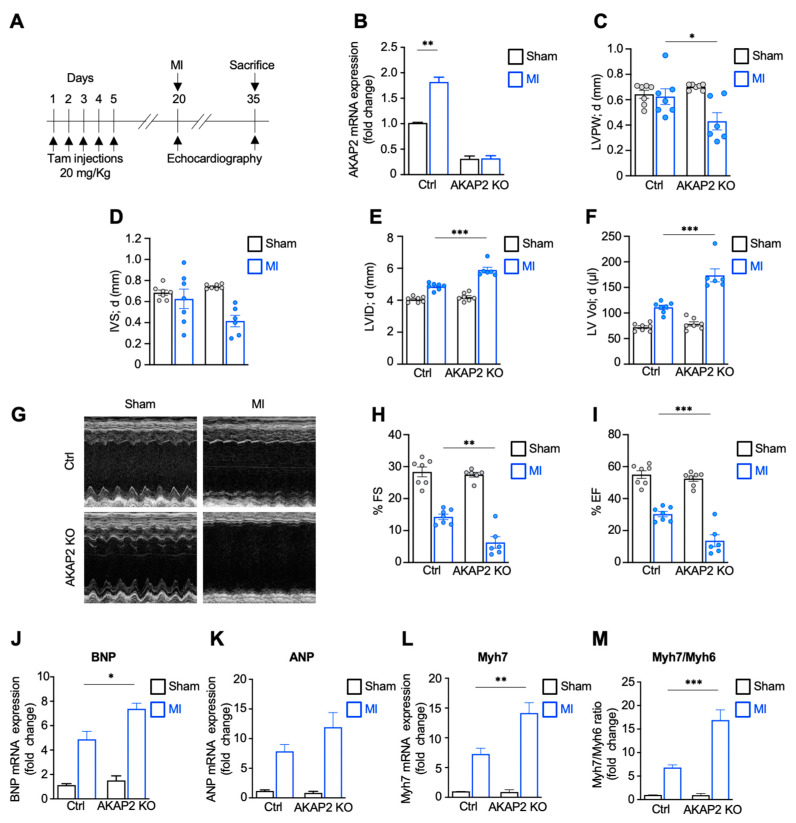
Targeted deletion of AKAP2 in adult cardiomyocytes exacerbates MI-induced cardiac stress and dysfunction. (**A**) Timeline of MI experimental protocol. MI was induced by LAD ligation 15 days after tamoxifen injections. Echocardiography was performed prior and 15 days after surgery. (**B**) AKAP2 mRNA expression in heart lysates from control (Ctrl) and AKAP2 KO male mice subjected to sham surgery or MI was assessed by quantitative real-time PCR. Ctrl-sham *n* = 4, Ctrl-MI *n* = 3, AKAP2 KO-sham *n* = 4, AKAP2 KO-MI *n* = 4. (**C**) End-diastolic (LVPW;d) left ventricular posterior wall thickness. (**D**) End-diastolic (IVS; d) intraventricular septum thickness. (**E**) End-diastolic (LVID; d) left ventricular internal diameter. (**F**) End-diastolic (LV Vol; d) left ventricular volume. (**G**) Transthoracic M-mode echocardiography tracings. (**H**,**I**) Fractional shortening (FS) and Ejection fraction (EF). Size of the experimental groups size for panels (**C**–**J**): Ctrl-sham *n* = 7, Ctrl-MI *n* = 7, AKAP2 KO-sham *n* = 7, AKAP2 KO-MI *n* = 6. (**J**–**M**) Quantitative real time PCR analysis of the expression of BNP (**J**), ANP (**K**), Myh7 (**L**). The Myh7: Myh6 ratio is shown **(M)**. Ctrl-sham *n* = 4, Ctrl-MI *n* = 4, AKAP2 KO-sham *n* = 4, AKAP2 KO-MI *n* = 4. Values are presented as mean ± SEM; * *p* < 0.05, ** *p* < 0.01 and *** *p* < 0.001.

To investigate whether cardiac AKAP2 plays a role in the adaptive response occurring after MI, 12-week-old male and female AKAP2 KO mice and their control littermates were subjected to MI or to sham operation. Echocardiographic analysis was performed before surgery and 2 weeks later (Figure 2A). AKAP2 KO in cardiac tissues was confirmed by qPCR (Figure 2B). Control MI mice developed the expected maladaptive response of the myocardium to the ligation of the left anterior descending coronary artery (LAD) and displayed left ventricular dilation (Supplementary Materials Figures S2E,F and S1C,D; Table 2) and reduced fractional shortening (FS) and ejection fraction (EF) (Figures S2G–I and S1E,F; Table 2).

Interestingly, AKAP2 KO mice subjected to LAD ligation displayed thinner left ventricular posterior wall (LVPW; d) (Figure 2C and Table 2) and interventricular septum (IVS; d) (Supplementary Materials Figure S1B; Table 2), and increased ventricular dilation (LVID; d; LV Vol; d) (Figure 2E,F and Supplementary Materials Figure S1C,D; Table 2) as compared to their control littermates. In addition, AKAP2 KO MI mice exhibited exacerbated cardiac dysfunction, as shown by significantly reduced FS and ejection fraction EF compared to control mice (Figure 2G–I and Supplementary Materials Figure S1E,F; Table 2).

We next assessed the impact of AKAP2 KO on the mRNA expression of molecular markers of cardiac stress in the infarct border zone. Our results indicate that the expression of *BNP, ANP* and *Myh7* genes was markedly elevated in AKAP2 KO mice compared to control animals (Figure 2J,L and Supplementary Materials Figure S1G–I). In line with these observations, the Myh7: Myh6 ratio was increased by approximately 2.5 folds both in male and female AKAP2 KO mice (Figure 2M and Supplementary Materials Figure S1J). Collectively, these findings suggest that AKAP2 KO in cardiomyocytes enhances the development of deleterious morphological and functional alterations in response to LAD ligation.

### 3.3. AKAP2 Knockout in Cardiomyocytes Enhances Myocardial Apoptosis, Fibrosis and Infarct Size after LAD Ligation

**Figure 3:**
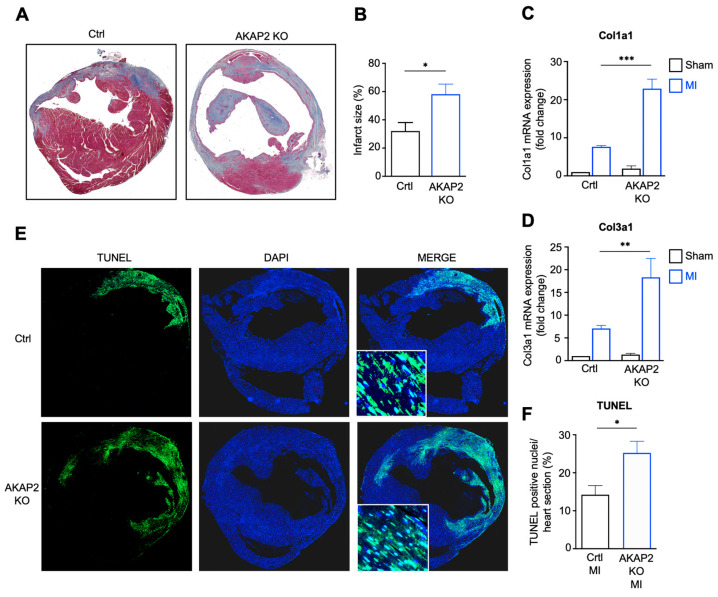
AKAP2 knockout in adult cardiomyocytes enhances myocardial apoptosis, fibrosis and infarct size after LAD ligation. (**A**) Masson’s Trichrome stainings performed on mid-ventricular heart sections from control (Ctrl) and cardiomyocyte-specific AKAP2 KO male mice 2 weeks after MI. (**B**) Quantitative assessment of infarct size. For each heart, the proportion of scar tissue in the left ventricle was quantified on 3 or 4 mid-ventricular transversal sections stained with Masson’s Trichrome. Ctrl-MI *n* = 7; AKAP2 KO-MI *n* = 4. (**C**,**D**) The relative mRNA expression of collagen 1a1 (**C**) and 3a1 (**D**) in heart lysates from the indicated mice groups was assessed by quantitative real-time PCR. Values were normalized to the expression of GAPDH. Ctrl-sham *n* = 4, Ctrl-MI *n* = 3, AKAP2 KO-sham *n* = 4, AKAP2 KO-MI *n* = 4. (**E**) Representative TUNEL and DAPI stainings of transversal heart sections taken from control (Ctrl) and cardiomyocyte-specific AKAP2 KO male mice 24 h after LAD ligation. (**F**) Quantification of apoptosis. The number of TUNEL-positive nuclei was normalized to the total number of nuclei per heart section. Data from 5 or 6 different heart sections were averaged for each mouse. Ctrl-MI *n* = 5; AKAP2 KO *n* = 7. Values are presented as mean ± SEM; * *p* < 0.05; ** *p* < 0.01; *** *p* < 0.001.

Based on our results suggesting that AKAP2 KO mice subjected to MI display worsened cardiac function, we next examined the impact of cardiac AKAP2 deletion on the infarct size measured 2 weeks after LAD ligation. Our results indicate that both male and female AKAP2 KO mice develop significantly larger infarcts compared to control littermates (Figure 3A,B and Supplementary Materials Figure S2A,B). This correlated with an increased expression of collagen type I alpha 1 (*Col1a1)* and collagen type III alpha 1 (*Col3a1*) genes in AKAP2 KO animals compared to control mice, as assessed by qPCR (Figure 3C,D and Supplementary Materials Figure S2C,D). To determine whether increased infarct size could derive from an increased myocardial cell death, we assessed the extent of apoptosis in control and AKAP2 KO mice 24 h after LAD occlusion. We could show that apoptosis, assessed by the number of TUNEL-positive nuclei per heart section, was increased by approximately 1.8 folds in male and 2.6 folds in female AKAP2 KO mice as compared to control animals (Figure 3E,F and Supplementary Materials Figure S2E,F). These findings suggest that AKAP2 deletion in cardiomyocytes results in enhanced myocardial apoptotic cell death in response to LAD occlusion, which is followed by the development of bigger infarcts. They also infer that AKAP2 exerts a protective function in cardiomyocytes during MI.

### 3.4. AKAP2 Assembles a Steroid-Receptor Coactivator-3-Based Signaling Complex Involved in Estrogen Receptor Activation

To get insights into the molecular mechanism whereby AKAP2 promotes protective effects in infarcted hearts, we initially performed a proteomic screen for AKAP2-interacting proteins expressed in cardiac tissues. To this end, AKAP2 was immunoprecipitated from whole heart lysates, and AKAP2 immunoprecipitates were subsequently analyzed by shotgun LC–MS/MS mass spectrometry.

**Figure 4:**
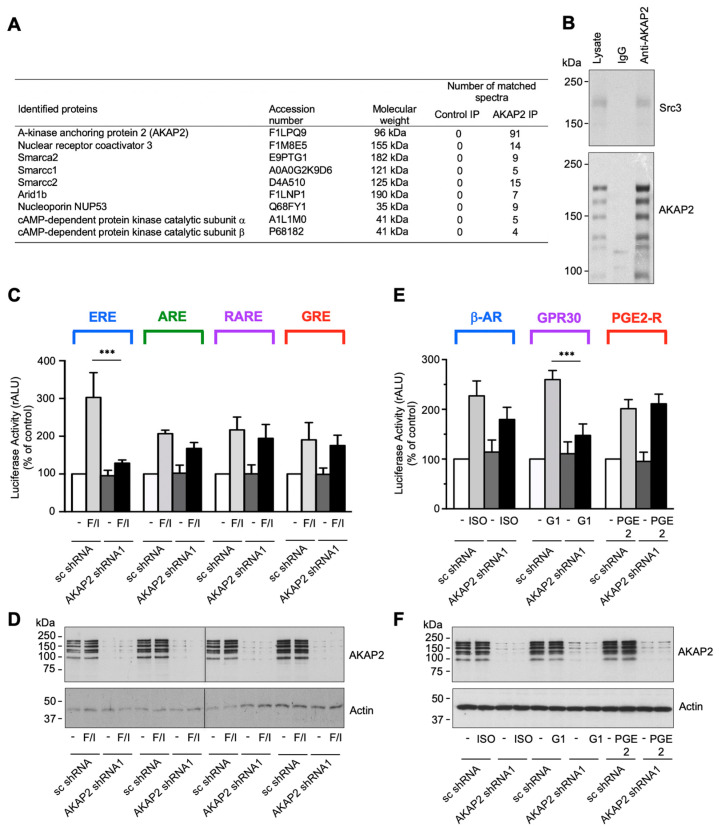
AKAP2 assembles a steroid-receptor coactivator-3-based signaling complex involved in estrogen receptor activation. (**A**) Complete list of proteins detected by shotgun LC–MS/MS mass spectrometry in AKAP2 immunoprecipitates from whole heart lysates. The number of matched spectra is reported for control and AKAP2 immunoprecipitations (IP). (**B**) Rat NVMs extracts were subjected to immunoprecipitation with either non-immune IgGs or affinity purified anti-AKAP-2 antibodies. Western blots of the immunoprecipitates and the cell extracts were revealed by using either anti-Src3 (upper panel), or affinity purified anti-AKAP2 polyclonal antibodies (lower panel). (**C**) Rat NVMs were infected by using lentiviruses encoding wild type (AKAP2 shRNA1) or scrambled (sc shRNA) AKAP2 shRNAs and subsequently transfected with ERE-, ARE-, RARE- or GRE-firefly luciferase reporter constructs. Seventy-two hours after infection, cells were incubated for 8 h in the absence or presence of 10^−5^ M FSK and IBMX. Firefly-luciferase activity was normalized to Renilla-luciferase activity. Results are the mean ± SE of three independent experiments; *** *p* < 0.011. (**D**) Expression of AKAP2 and actin in the lysates was assessed by immunoblot blot, using specific antibodies, as indicated. (**E**) Rat NVMs were infected as indicated in (**B**) and subsequently transfected with ERE-firefly luciferase and *Renilla* luciferase reporter constructs. Seventy-two hours after infection cells were incubated for 8 h in the absence or presence of 10^−6^ M ISO, 10^−7^ M G1, or 10^−7^ M PGE2. Firefly-luciferase activity was normalized to Renilla-luciferase activity. Results are the mean ± SE of three independent experiments; *** *p* < 0.01. (**F**) Expression of AKAP2 and actin in the lysates was assessed as indicated in (**D**).

This approach identified peptides from several AKAP2 associated proteins, including transcriptional regulators and nuclear proteins, such as the steroid receptor coactivator 3 (Src3), catalytic and accessory subunits of the SWI/SNF complex (Smarca2, Smarcc1 and Smarcc2) the nuclear AT-rich interactive domain-containing protein 1b (Arid1b), and nucleoporin 35 (NUP35) (Figure 4A). The mass spectrometry proteomics data were deposited to the ProteomeXchange Consortium via the PRIDE partner repository. Among the identified AKAP2 interacting proteins, Src-3 attracted our attention because of its potential of regulating transcriptional responses promoting cell survival [39]. The interaction between AKAP2 and Scr-3 was confirmed in rat NVMs when endogenous Src-3 was detected in AKAP2 immunoprecipitates (Figure 4B, upper panel).

Src-3 belongs to the p160 family of transcriptional co-activators shown to be involved in the regulation of several nuclear receptors (NRs), including glucocorticoid receptors (GRs), estrogen receptors (ERs), retinoid receptors (RARs and RXRs) and peroxisome proliferator-activated receptors (PPARs) [40]. Based on this evidence we raised the hypothesis that AKAP2 might target PKA and Src-3 to specific nuclear receptors to promote their activation in response to signals that raise the intracellular cAMP concentration.

To test this possibility, we initially investigated whether AKAP2 silencing in rat NVMs could influence the ability of forskolin (FSK) and 3-isobutyl-1-methylxanthine (IBMX), which activate cAMP signaling globally, to enhance the activity of ERs, androgen receptors (ARs), RARs and GRs. These NRs were selected based on their known ability to be regulated by both PKA and Src-3 [40,41,42,43,44]. Rat NVMs were infected by using lentiviruses encoding AKAP2 specific shRNAs or scrambled control shRNAs (sc shRNA) and subsequently transfected with vectors containing the Firefly luciferase reporter under the control of specific NR responsive elements. Our results indicate that stimulation of rat NVMs expressing sc-shRNAs, using FSK and IBMX, results in the activation of all four tested nuclear receptors. Interestingly, AKAP2 silencing suppressed cAMP-induced activation of ER without significantly affecting the activity of ARs, RARs and GRs (Figure 4C). AKAP2 silencing was confirmed by immunoblot (Figure 4D). These results suggest that, in cardiomyocytes, AKAP2 mediates the activation of ERs induced by cAMP.

We next sought to determine which G-protein-coupled receptor (GPCR) expressed in cardiomyocytes promotes the activation of the AKAP2–PKA–ER signaling pathway. To address this question, we investigated the role of β-ARs, GPR30 and PGE2 receptors (PGE2-Rs). These Gs-coupled receptors have been shown to promotes protective signaling in cardiomyocytes and could therefore represent potential upstream activators of the AKAP2 complex [45,46,47,48]. Cardiomyocytes expressing sc shRNAs or AKAP2 specific sRNAs were incubated in the absence or presence of the β-adrenergic agonist isoproterenol (ISO), the selective GPR30 agonist G1, or PGE2, and ER activation measured by monitoring the activity the firefly luciferase reporter. Our results indicate that AKAP2 silencing (Figure 4F) significantly reduces G1-induced ER activation without influencing the responses to ISO and PGE2 (Figure 4E). To exclude the possibility that that inhibition of ER signaling was due to off-target effects of the shRNA targeting AKAP2, we confirmed these findings by using a second independent shRNA (Supplementary Materials Figure S3A).

We could further show that incubation of cardiomyocytes with cell-permeable type II PKA- anchoring disrupting peptides (SuperAKAP-IS) but not with control scrambled peptides (Sc-SuperAKAP-IS) or type I PKA-anchoring inhibitory peptides (RIAD) inhibited the ability of GPR30 to promote ER transcriptional activity, thus confirming the implication of anchored PKA in ER regulation (Supplementary Materials Figure S3B). A similar inhibitory effect could be observed by incubating cardiomyocytes with the cell-permeable PKA inhibitor Myr-PKI (Supplementary Materials Figure S3C). Collectively, these results suggest that AKAP2 and anchored type II PKA mediate GPR30-induced stimulation of ER activity in cardiomyocytes.

### 3.5. AKAP^2^ Favors PKA-Mediated Phosphorylation of ERα

**Figure 5:**
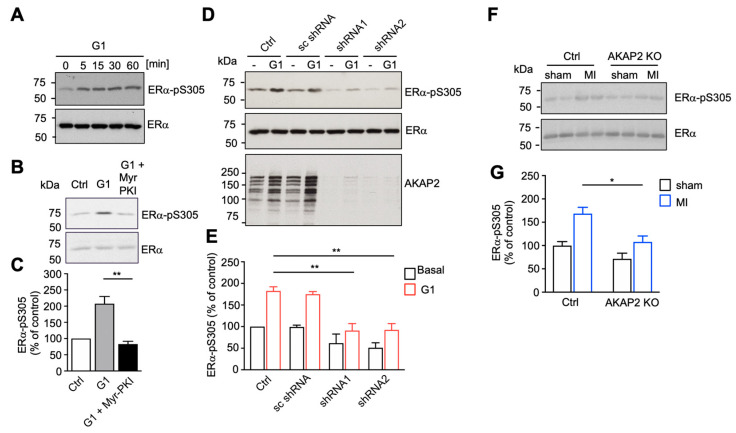
AKAP2 favors PKA-mediated phosphorylation of ERa. (**A**) Rat NVMs were serum starved for 24 h and subsequently treated with 10^−7^ M G1 for the indicated periods of time. Phosphorylation of S305 on ERα was revealed by Western blot, using phospho-specific antibodies. (**B**,**C**) Rat NVMs were serum starved for 24 h and subsequently treated with or without 10^−7^ M G1 for 15min in the presence or absence of 10−6 M Myr-PKI. Phosphorylation of S305 on ERα was revealed by using phospho-specific antibodies. The amount of phospho-ERα was normalized to the total amount of ERα. Results are expressed as mean ± SE of 3 experiments; ** *p* < 0.01. (**D**,**E**) Rat NVMs were infected by using control lentiviruses or lentiviruses encoding wild type (AKAP2 shRNA 1 and 2) or scrambled (sc shRNA) AKAP2 shRNAs. Cells were serum starved for 24 h and subsequently treated with or without 10^−7^ M G1 for 15min. Phosphorylation of S305 on ERα was revealed and quantified as indicated in (**B**,**C**). Expression of AKAP2 in the lysates was assessed by immunoblot, using specific antibodies. Results are expressed as mean ± SE of 3 experiments; ** *p* < 0.01. (**F**,**G**) Cardiac lysates were prepared from control (Ctrl) or cardiomyocyte-specific AKAP2 KO mice 24 h after sham operation or LAD ligation (MI). Phosphorylation of ERα S305 was assessed by Western blot and quantified as indicated in (**B**,**C**). Ctrl-sham *n* = 4, Ctrl-MI *n* = 4, AKAP2 KO-sham *n* = 4, AKAP2 KO-MI *n* = 4; * *p* < 0.05.

It is well established that ERα can be activated in an agonist-independent manner through PKA-mediated phosphorylation of serine 305 (S305) [44,49]. Based on this evidence, we investigated the possibility that AKAP2 might favor PKA-mediated phosphorylation of ERα in response to GPR30 stimulation. We could initially show that stimulation of rat NVMs with 10−7 M G1 results in the time-dependent phosphorylation of S305 on ERα as shown by immunoblot, using anti-phosphospecific antibodies (Figure 5A). G1-induced ERα phosphorylation was PKA-dependent since it was prevented by Myr-PKI (Figure 5B,C). Importantly, the ability of GPR30 receptors to promote S305 phosphorylation in rat NVMs was abolished, following silencing of AKAP2, using two independent shRNAs (Figure 5D,E). Taken together, these results suggest that AKAP2-anchored PKA mediates GPR30-induced ERα phosphorylation and activation in cardiomyocytes.

To assess whether AKAP2 controls ERα phosphorylation also in infarcted hearts, we measured the levels of phospho-S305 in control and AKAP2 KO animals, 24 h after LAD ligation or sham operation. Our results indicate that LAD ligation increases the phosphorylation of S305 by 1.6 folds in the heart of control mice (Ctrl) (Figure 5F,G). This effect was significantly reduced in AKAP2 KO hearts, suggesting that AKAP2 mediates ERα phosphorylation in response to MI (Figure 5F,G).

### 3.6. AKAP2 Promotes ERα-Mediated Regulation of Bcl2 and VEGFa in Cardiomyocytes

**Figure 6:**
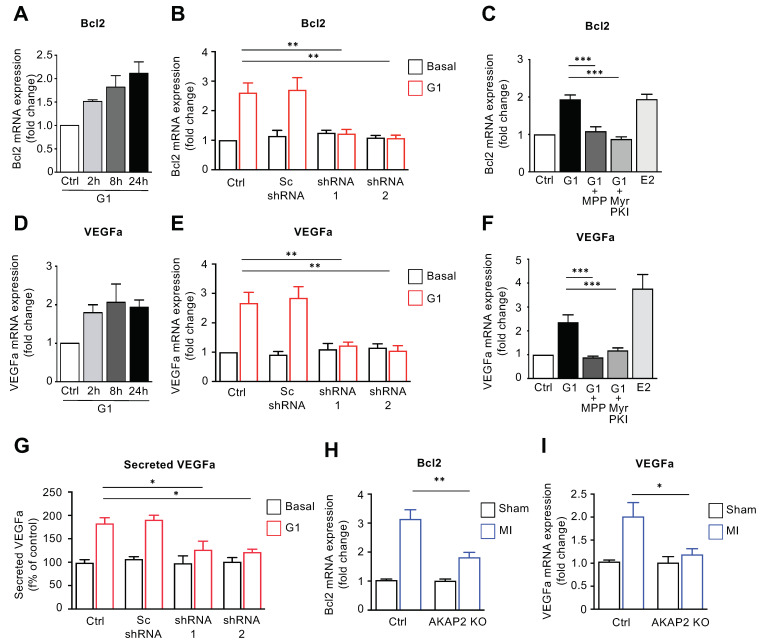
AKAP2 promotes ERα-mediated regulation of Bcl2 and VEGFa in cardiomyocytes. (**A**,**D**) Time course of G-1-induced Bcl2 (**A**) and VEGFa (**D**) mRNA expression. Rat NVMs were serum starved for 24 h and subsequently treated with 10^−7^ M G1 for the indicated periods of time. Bcl2 and VEGFa mRNA expression was assessed by quantitative real-time PCR. (**B**,**E**) Rat NVMs were infected by using control lentiviruses or lentiviruses encoding wild type (AKAP2 shRNA 1 and 2) or scrambled AKAP2 shRNAs (sc shRNA). Seventy-two hours after infection, cells were serum-starved for 24 h and subsequently treated with 10^−7^ M G1 for 24 h. Bcl2 and VEGFa mRNA expression was determined by quantitative PCR. Results are expressed as mean ± SE of 3 experiments. (**C**,**F**) Rat NVMs were serum starved for 24 h and subsequently left untreated (Ctrl) or treated with 10^−7^ M G1 in the absence or presence of 10^−5^ M MPP or Myr-PKI. Control stimulations with 10−7 M 17β-estradiol (E2) were performed to promote direct ER activation. Bcl2 and VEGFa mRNA expression was assessed by quantitative real-time PCR. (**G**) Rat NVMs were infected and treated as indicated in (**B**). VEGFa amounts in culture media was assessed by ELISA. Results are expressed as mean ± SE of 3 experiments. (**H**,**I**) Cardiac lysates were prepared from control (Ctrl) or cardiomyocyte-specific AKAP2 KO mice 2 weeks after sham operation or LAD ligation (MI). Bcl2 and VEGFa mRNA expression in cardiac lysates was assessed by quantitative PCR. Ctrl-sham *n* = 4, Ctrl-MI *n* = 4, AKAP2 KO-sham *n* = 4, AKAP2 KO-MI *n* = 4; * *p* < 0.05, ** *p* < 0.01 and *** *p* < 0.001.

Based on our results showing that cardiac AKAP2 exerts a protective action in hearts subjected to MI, we investigated whether the GPR30–AKAP2–PKA signaling pathway could promote the transcriptional activation of ER-dependent genes involved in cardioprotection. We focused our interest on genes displaying cardiac expression and the ability to be regulated by ERα (21, 28, 32). Based on these criteria we considered the following genes: vascular endothelial growth factors A and B (*Vegfa* and *Vegfb),* the anti-apoptotic protein B-cell lymphoma 2 (*Bcl2)*, cyclin D1 (*Ccnd1*) and nitric oxide synthase 1 (*Nos1)*. Among these, *Vegfb* was not upregulated following stimulation of rat NVMs with G1 for 2, 8 or 24 h (Supplementary Materials Figure S4A). Similarly, AKAP2 silencing did not influence *Vegfb* expression (Supplementary Materials Figure S4B). Two additional genes, *Ccnd1* and *Nos1*, were activated by G1 in an AKAP2-independent manner as silencing of the anchoring protein did not significantly reduce their expression level (Supplementary Materials Figure S4C–F). Finally, AKAP2 downregulation resulted in a significant inhibition of G1-induced transcriptional of activation *Vegfa* and *Bcl2* genes (Figure 6A,B,D,E). In control experiments we could show that incubation of rat NVMs, using Myr-PKI or the ERα selective inhibitor MPP abolishes G1-induced *Vegfa* and *Bcl2* expression confirming that GPR30-mediated upregulation of *Vegfa* and *Bcl2* mRNAs requires both functional PKA and ERα (Figure 6C,F). ELISA assays performed by using culture media isolated from control and AKAP2-silenced rat NVMs indicate that downregulation of AKAP2 expression also inhibits G1-induced Vegfa secretion (Figure 6G). Importantly, our results point out that AKAP2 controls *Vegfa* and *Bcl2* gene transcription also in infarcted hearts, as shown by the fact that, 2 weeks after LAD ligation, AKAP2 KO hearts display reduced *Vegfa* and *Bcl2* mRNA levels compared to control mice.

Collectively, these findings suggest that AKAP2 mediates the activation of a subset of anti-apoptotic (*Bcl2*) and pro-angiogenic (*Vegfa*) ERα-regulated genes, both in isolated cardiomyocytes and in remodeling hearts. In this respect, the ability of AKAP2 to modulate the expression of *Bcl2* is in line with our current finding showing that AKAP2 limits the extent of cardiac apoptosis after LAD ligation (Figure 3E,F), whereas AKAP2-mediated transcriptional activation of the *Vegfa* gene suggests a possible role of the anchoring protein in modulating angiogenesis.

### 3.7. The AKAP2–Src3 Complex Mediates GPR30-Induced ER Activation in Cardiomyocytes

**Figure 7:**
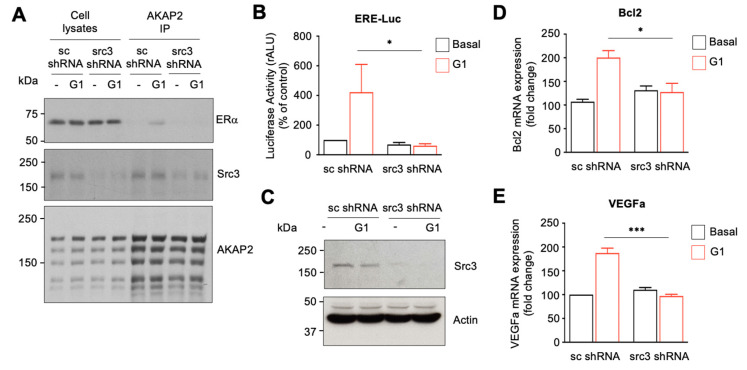
GPR30-induced ER activation in cardiomyocytes requires Src3. (**A**) Rat NVMs were infected by using lentiviruses encoding Src3 shRNAs or scrambled shRNAs (sc shRNA). Seventy-two hours after infection, cells were serum starved for 24 h and subsequently incubated with or without 10^−7^ M G1 for 15 min. Rat NVMs were then lysed and extracts subjected to immunoprecipitation with affinity purified anti-AKAP-2 antibodies. Western blots of the immunoprecipitates and the cell extracts were revealed by using either anti-ERα (upper panel), anti-Src3 (middle panel) or anti-AKAP-2 monoclonal antibodies (lower panel). (**B**) Rat NVMs were infected by using lentiviruses encoding Src3 or scrambled (sc) shRNAs and subsequently transfected with ERE-firefly luciferase and renilla luciferase reporter constructs. Seventy-two hours after infection, cells were incubated for 8 h in the absence or presence of 10^−7^ M G1. Firefly-luciferase activity was normalized to Renilla-luciferase activity. Results are the mean ± SE of 6 independent experiments; * *p* < 0,05. (**C**) Expression of src3 and actin in the lysates was assessed as indicated by immunoblot, using specific antibodies. (**D**,**E**) Rat NVMs were infected as indicated in (**A**). Seventy-two hours after infection, cells were serum starved for 24 h and subsequently treated with 10^−7^ M G1 for 24 h. Bcl2 and VEGFa mRNA expression was determined by quantitative PCR. Results are the mean ± SE of 3 independent experiments; * *p* < 0,05; *** *p* < 0,001.

Based on our results showing that the transcriptional co-activator Src3 forms a complex with AKAP2 and on the evidence that AKAP2 mediates ERα activation in cardiomyocytes, we raised the question of whether Src3 could mediate the recruitment of Erα to the AKAP2 signaling complex. To address this point, we determined the ability of ERα to co-immunoprecipitate with AKAP2 from control and Src3-silenced cardiomyocytes incubated with or without G1. Our results indicate that stimulation of GPR30 promotes the association between AKAP2 and ERα and that silencing of Src3 abolishes the ability of ERα to co-immunoprecipitate with AKAP2 (Figure 7A). This suggests that the formation of the AKAP2–ERα complex is dynamically regulated by GPR30 receptors and mediated by Src3.

We next assessed the implication of Scr3 in regulating ERα activity in rat NVMs. To address this question, we monitored the ability of G1 to induce activation of an ER-activated Luciferase reporter in control or Src3-silenced cardiomyocytes. Our results indicate that Src3 downregulation abolished G1-induced ERα transcriptional activity (Figure 7B). In line with these findings Scr3 silencing strongly reduced *Bcl2* and *Vegfa* upregulation induced following GPR30 activation (Figure 7D,E). Src3 silencing was confirmed by immunoblot (Figure 7C). Taken together, these results suggest that, in cardiomyocytes, GPR30-induced expression of cardioprotective *Vegfa*, and *Bcl2* requires both AKAP2 and Src3.

### 3.8. AKAP2 Knockout in Adult Cardiomyocytes Reduces Vessel Number in Infarcted Hearts

**Figure 8:**
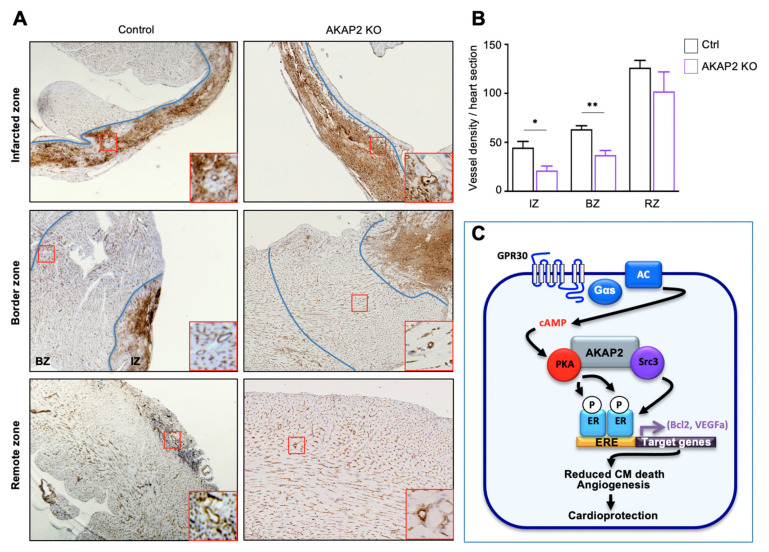
AKAP2 knockout in adult cardiomyocytes reduces vessel number in infarcted and border zones. (**A**) Control (Ctrl) or cardiomyocyte-specific AKAP2 KO mice underwent sham operation or LAD ligation. Two weeks after surgery, hearts were harvested and included in paraffin. Deparaffinized-heart sections were stained for lectin. (**B**) For each heart, the number of vessels was quantified on 3 or 4 heart sections, using the ImageJ software. Vessel density was reported for infarcted (IZ), border (BZ) and remote zones (RZs). Results are presented as the mean ± SEM. Ctrl-MI *n* = 7, AKAP2 KO-MI *n* = 4; * *p* < 0,05; ** *p* < 0,01. (**C**) AKAP2 assembles a signaling complex in cardiomyocytes, which coordinates the ERα activating activity of PKA and Src3. The AKAP2-anchored pool of PKA becomes activated in infarcted hearts and promotes the phosphorylation of ERα on Ser305. ERα phosphorylation in conjunction with Src3, enhances the transcriptional activity of ERα, which increases the transcription of anti-apoptotic and pro-angiogenic genes, such as Bcl2 and VEGFa. Consequently, apoptosis is reduced, and the number of blood vessels increased in the infarcted region, which protects the heart during ischemic stress.

*Vegfa* is a pro-angiogenic gene shown to be induced after MI and to be associated with reduced collagen deposition and infarct size, and improved cardiac function, by promoting the formation of new vessels [50,51]. Based on these findings, we next examined the impact of knocking-out AKAP2 in cardiomyocytes of male and female mice on cardiac vessel density two weeks post-MI. To visualize the vasculature, transversal heart sections were stained for endothelial lectin [50]. No detectable differences were observed in left ventricular vessel density between sham operated WT and AKAP2 KO animals. However, two weeks post-MI, the vessel density was significantly decreased both in the infarcted (IZ) and border zone (BZ) of male and female AKAP2 KO mice compared to control littermates (Figure 8A,B and Supplementary Materials Figure S5A,B). The remote zone (RZ) had no notable difference in the number of vessels (Figure 8A,B and Supplementary Materials Figure S5A,B). Taken together, these results indicate that AKAP2 is implicated in the induction of *Vegfa*, in cardiomyocytes, and that the deletion of this anchoring protein reduces vessel number in infarcted hearts.

## 4. Discussion

In industrialized countries, up to 70% of all heart-failure syndromes arise from prior coronary artery disease and myocardial infarction [52]. In this context, identifying the molecular determinants that favor cardiomyocyte protection and survival in infarcted hearts is key for deciphering the intrinsic mechanisms affecting post-MI cardiac remodeling and development of heart failure. 

In the present study, we showed that AKAP2 assembles a cardioprotective signaling complex that limits remodeling and cardiac dysfunction after MI. This transduction unit, which contains PKA and the transcriptional regulator Src3, recruits and activates Erα in cardiomyocytes in response to the activation of GPR30 (Figure 8C). In particular, our findings indicate that GPR30-induced activation of AKAP2-anchored PKA leads to the phosphorylation of ERα on Ser305 (Figure 8C). This phosphorylation event enhances the transcriptional activity of ERα, which leads to the upregulation of a subset of anti-apoptotic and pro-angiogenic genes, including *Bcl2* and *VEGFa* (Figure 8C). In line with these findings, AKAP2 KO in infarcted hearts leads to a reduction in *Bcl2* and *VEGFa* expression, which is associated with an increase in myocardial apoptosis and a decrease in blood vessel number. These events lead to an increase in infarct size and exacerbated cardiac dysfunction. Overall, these findings provide evidence that AKAP2 coordinates cardioprotective signals that enhance the activity of ERα to reduce apoptosis and promote vessel formation in MI-injured hearts (Figure 8C).

The fact that AKAP2 KO in cardiomyocytes negatively impacts cardiac function in MI mice without inducing deleterious effects in sham operated animals suggests that this cardioprotective anchoring protein is mainly mobilized in stressed hearts undergoing MI and does not contribute, in the short-term, to preserve baseline cardiac function (Figure 1, Figure 3 and Figure 6 and Supplementary Materials Figure S5). This is consistent with our observation that AKAP2 is rapidly upregulated after LAD ligation (Figure 1). However, since our functional evaluation of the impact of cardiac AKAP2 KO was carried out over a period of only a few weeks, we cannot rule out the possibility that AKAP2 KO might induce alterations of baseline cardiac function that might become apparent only in the long-term, when mice get older.

In AKAP2 KO mice, the first signs of myocardial alterations, including increased myocardial apoptosis, appear already 24 h after LAD ligation (Figure 2). This suggest that AKAP2 contributes to an early protective response that limits cell death and MI expansion (Figure 3). Interestingly, AKAP2 upregulation is maintained up to several days after infarction suggesting that this anchoring protein could also exert a prolonged protective action in stressed hearts (Figure 1A–C). In line with this assumption, AKAP2 KO leads to a downregulation of Bcl2 and Vegfa gene expression, which is still detectable 2 weeks after surgery (Figure 6H,I) and which is associated with decreased blood vessels’ density in the IZ and BZ, increased infarct size and reduced EF and FS (Figure 2, Figure 3 and Figure 8). 

The rapid mobilization of the AKAP2/PKA/Src3 signaling complex in stressed cardiomyocytes, and the consequent activation of ERα can be viewed as a key intrinsic cardioprotective mechanism that reduces the deleterious functional consequences of MI. This contrasts with what has been shown for other AKAPs expressed in the heart, including AKAP6 and phosphatidyl inositol 3 kinase γ (PI3Kγ), whose upregulation in response to stress leads to cardiac remodeling and heart failure [22,23]. Moreover, in contrast to what is observed for AKAP2, several cytoprotective AKAPs expressed in cardiomyocytes are downregulated in response to cardiac stress. Indeed, AKAP1, an anchoring protein involved in the maintenance of mitochondrial function and integrity, is rapidly proteolyzed in infarcted hearts, which contributes to MI-induced oxidative stress and cardiac dysfunction [30]. Similarly, a significant reduction of AKAP5 and AKAP12 expression has been shown to occur in remodeling hearts in response to pressure overload and angiotensin II (Ang-II) exposure [25]. Downregulation of AKAP5 leads to alterations in calcium cycling and depressed cardiac function, whereas the reduction of AKAP12 levels is linked to oxidative stress and inflammation [25,28]. Therefore, it appears that AKAP2 is the only known AKAP mobilized after MI that can confer cardioprotection. Based on this consideration, the use of upstream inducers of AKAP2 expression and signaling (e.g., GPR30 agonists) could be investigated as a potential approach limiting the detrimental functional consequences of MI. 

Our results suggest that AKAP2 favors PKA-mediated phosphorylation and transcriptional activation of ERα in response to the stimulation of GPR30 but not of other Gs-coupled GPCRs, such as β-ARs and PGE2-Rs (Figure 4C,E). The molecular basis of the selective functional coupling between GPR30 and AKAP2 remains to be investigated. One possible hypothesis is that GPR30, β-ARs and PGE2-Rs promote the formation cAMP microdomains that are physically and/or functionally distinct [53]. In this configuration, a physical proximity between AKAP2 and GPR30 might ensure that AKAP2-anchored PKA is accessed only by cAMP generated locally in response to receptor stimulation. In support of the notion that different GPCRs can initiate diverse cAMP responses, β-ARs and PGE2 EP_4_ receptors have been shown to be located in separate membrane domains and induce distinct cAMP signals in cardiomyocytes [54]. 

ΕRα can be activated either through direct estrogen binding or in an agonist-independent manner through phosphorylation [44,55]. In particular, studies performed in breast cancer cells indicate that phosphorylation of serine 305 in the hinge region of ERα by PKA increases receptor transcriptional activity [49,56]. The ability of AKAP2 to selectively control PKA-mediated activation of ERα without influencing the activity of ARs, RARs and GRs (Figure 4C) could be linked to its capacity to physically interact with ERα. Indeed, our results indicate that AKAP2 and ΕRα form a signaling complex whose assembly is enhanced following GPR30 activation (Figure 7A). The fact that the downregulation or knockout of AKAP2 expression completely suppresses PKA-mediated activation (Figure 4 and Figure 5) of ERa indicates that AKAP2 is the main anchoring protein targeting PKA to ERα in cardiomyocytes. In this respect, previous findings suggest that PKA-induced phosphorylation of ERα in breast cancer cells is regulated by AKAP-Lbc [57]. This raises the hypothesis that, in cardiomyocytes and breast cancer cells, ERα phosphorylation might be controlled by different pools of PKA anchored by distinct anchoring proteins. 

Our findings underscore the role of GPR30-dependent cAMP signaling in activating ERα-mediated genomic responses (Figure 5E and Figure 6). Crosstalk between membrane estrogen receptors and nuclear estrogen receptors has been previously observed in various cells and tissues [58,59]. In particular, studies performed on human cancer cell lines have shown that the transcriptional responses induced by GPR30 are largely mediated by nuclear estrogen receptors [60]. So far, the molecular mechanisms underlying this crosstalk have not been precisely characterized. Here, we propose a central role for AKAP2, PKA and Src3 in conveying signals from membrane to nuclear ERs. Mechanistically, Src3 could mediate this effect by promoting the recruitment of ERα to AKAP2 (Figure 7A) in response to GPR30 stimulation, thus favoring PKA-mediated phosphorylation and activation of the receptor (Figure 5). Interestingly, phosphorylation of serine 305 on unligated ΕRα has been shown to significantly increase its interaction with Src3 [56], which could further enhance ERα activity. Based on these observations, the AKAP2–Src3 signaling complex can be viewed as a molecular coordinator that synchronizes the GPR30/cAMP/PKA pathway with ERα activation in cardiomyocytes.

Our findings that AKAP2, PKA and Src3 cooperate to promote the activation of cardioprotective responses are consistent with recent findings showing that a direct activator of steroid receptor co-activators known as MCB-613 reduces cardiac remodeling and preserves heart function after MI by reducing cardiomyocyte apoptosis, infarct size and fibrosis [39]. Based on this evidence, Src family members can be considered as mediators of protection and repair in infarcted hearts.

Our results indicate that AKAP2 confers protection against MI-induced cardiac dysfunction both in female and male mice (Figure 2 and Figure 3; Supplementary Materials Figures S1 and S2). These findings are in line with previous observations that GPR30, which we identified as an upstream activator of the AKAP2–Src3–PKA signaling axis, confers protection against cardiac ischemia-reperfusion injuries in both genders [61,62,63]. While it is well established that GPR30 functions as a membrane estrogen receptor, recent studies indicate that several molecules, including estrogenic derivatives and plant-derived polyphenolic compounds, can act as GPR30 agonists [64], thus suggesting that this receptor can be activated by a variety of ligands. In this respect, the endogenous agonists activating GPR30 in female and male infarcted hearts are currently unknown. Identifying such endogenous activators will be a crucial challenge, since it could open new venues in the treatment of MI-associated remodeling and dysfunction. 

In conclusion, our current findings have several implications. Firstly, they provide evidence that AKAP2, an anchoring protein with a previously unknown implication in cardiac remodeling, is rapidly upregulated in the myocardium in response to left anterior descending artery (LAD) ligation and confers protection against MI expansion and cardiac dysfunction. Secondly, they highlight that AKAP2 assembles a signaling complex in cardiomyocytes that coordinates the action of PKA and Src3 to modulate the function of ERα, and, consequently, the induction of anti-apoptotic and pro-angiogenic genes. Thirdly, they indicate that targeted deletion of the AKAP2 gene in adult cardiomyocytes increases myocardial apoptosis, reduces new vessel formation and exacerbates cardiac dysfunction associated with MI, thus suggesting that AKAP2 functions as a coordinator of cardioprotective signals that attenuate myocardial damage in infarcted hearts.

## Figures and Tables

**Table 1:** Physiological and echocardiographic parameters in control and AKAP2 KO mice at baseline.

	Males		Females	
	Control	AKAP2 KO	Control	AKAP2 KO
n	27	32	29	20
Heart Rate (beads/min)	470 ± 8	499 ± 11	477 ± 9	490 ± 14
Body Weight (g)	29.43 ± 1.00	28.41 ± 0.69	22.46 ± 0.31	22.71 ± 0.45
LVPW; d (mm)	0.66 ± 0.01	0.69 ± 0.01	0.66 ± 0.01	0.69 ± 0.02
LVPW; s (mm)	0.88 ± 0.02	0.95 ± 0.03	0.92 ± 0.02	0.94 ± 0.03
IVS; d (mm)	0.68 ± 0.01	0.71 ± 0.01	0.69 ± 0.01	0.72 ± 0.03
IVS; s (mm)	1 ± 0.02	1.03 ± 0.02	1.01 ± 0.02	1.02 ± 0.03
LVID; d (mm)	4.28 ± 0.05	4.15 ± 0.12	3.87 ± 0.06	3.95 ± 0.08
LVID; s (mm)	3.29 ± 0.07	3.16 ± 0.11	2.86 ± 0.08	2.94 ± 0.09
LV Vol.; d (µL)	82.66 ± 2.3	77.4 ± 4.84	65.35 ± 2.43	68.95 ± 3.09
LV Vol.; s (µL)	44.65 ± 2.02	41.27 ± 3.41	32.25 ± 2.07	34.37 ± 2.61
FS (%)	23.19 ± 0.99	24.05 ± 0.93	26.45 ± 1.23	25.82 ± 1.34
EF (%)	46.39 ± 1.62	47.91 ± 1.6	51.87 ± 1.87	50.86 ± 2.12

LVPW; d and LVPW; s = left ventricular posterior wall thickness during diastole and systole. IVS; d and IVS; s = interventricular septum thickness during diastole and systole. LVID; d and LVID; s = left ventricular internal diameter during diastole and systole. LV vol.; d and LV vol.; s = left ventricular volume during diastole and systole. FS, fractional shortening; EF, ejection fraction. Values are presented as mean ± SEM.

**Table 2:** Physiological and echocardiographic parameters in control and AKAP2 KO mice two weeks post MI or sham surgery.

	Control		AKAP2 KO	
	Sham	2 Weeks Post-MI	Sham	2 Weeks Post-MI
Male mice				
n	7	7	7	6
Heart Rate (beats/min)	515 ± 18	509 ± 37	563 ± 32	537 ± 38 ns
Body Weight (g)	29.23 ± 1.12	27.82 ± 1.13	29.57 ± 1.11	28.37 ± 0.83 ns
LVPWd (mm)	0.64 ± 0.03	0.62 ± 0.06	0.70 ± 0.01	0.43 ± 0.07 *
IVSd (mm)	0.68 ± 0.03	0.63 ± 0.09	0.74 ± 0.01	0.42 ± 0.05 ns
LVIDd (mm)	4.05 ± 0.07	4.86 ± 0.08	4.19 ± 0.09	5.89 ± 0.18 ***
LV Volume d (µl)	72.19 ± 2.82	111.10 ± 4.14	78.66 ± 4.16	173.60 ± 12.67 ***
%FS	28.35 ± 1.54	14.30 ± 0.87	26.73 ± 0.98	6.30 ± 1.81 **
%EF	55.03 ± 2.42	30.30 ± 1.65	52.48 ± 1.62	13.65 ± 3.73 ***
Female mice				
n	8	6	6	8
Heart Rate (beats/min)	509 ± 20	505 ± 37	576 ± 12	519 ± 24 ns
Body Weight (g)	22.91 ± 0.62	22.23 ± 0.58	21.62 ± 0.91	23.18 ± 0.44 ns
LVPWd (mm)	0.63 ± 0.02	0.65 ± 0.08	0.68 ± 0.03	0.68 ± 0.14 ns
IVSd (mm)	0.67 ± 0.01	0.61 ± 0.02	0.75 ± 0.03	0.37 ± 0.03 ***
LVIDd (mm)	3.81 ± 0.09	4.12 ± 0.17	3.76 ± 0.05	5.04 ± 0.23 **
LV Volume (µl)	62.88 ± 3.55	79.93 ± 8.60	60.47 ± 2.04	122.90 ± 13.19 *
%FS	27.74 ± 1.21	16.89 ± 3.83	32.55 ± 2.76	7.30 ± 1.26 *
%EF	54.37 ± 1.87	34.89 ± 7.16	61.11 ± 4.26	16.07 ± 2.68 *

LVPW; d = left ventricular posterior wall thickness during diastole. IVS; d = interventricular septum thickness during diastole. LVID; d = left ventricular internal diameter during diastole. LV vol.; d = left ventricular volume during diastole. FS, fractional shortening; EF, ejection fraction. Values are presented as mean ± SEM; * *p* < 0.05, ** *p* < 0.01 and ** *p* < 0.001 as compared to values measured in control mice 2 weeks post-MI.

## Data Availability

The mass spectrometry proteomics data were deposited into the ProteomeXchange Consortium via the PRIDE partner repository with the dataset identifier PXD028514 and 10.6019/PXD028514. Additional data needed to evaluate the conclusions in the paper are present in the Supplementary Materials. Further information and requests for reagents may be addressed to, and will be fulfilled by, the corresponding author (D.D.) (dario.diviani@unil.ch).

## Supplementary Materials

The following are available online at https://www.mdpi.com/article/10.3390/cells10112861/s1. Figure S1: Targeted deletion of AKAP2 in adult cardiomyocytes exacerbates MI-induced cardiac dysfunction in female mice. Figure S2: AKAP2 knockout in adult cardiomyocytes enhances myocardial apoptosis, fibrosis and infarct size in female mice after LAD ligation. Figure S3: AKAP2 mediates GPR30-induded ER-activation in cardiomyocytes. Figure S4: AKAP2 does not participate in the regulation of VEGFb, Cyclin D1 and Nos1. Figure S5: AKAP2 knockout in adult cardiomyocytes reduces vessel number in infarcted and border zones of female mice.
