# Supplementary material for: A-Kinase Anchoring Protein 2 Promotes Protection against Myocardial Infarction
Source: Cells. 2021 Oct 23;10(11):2861. doi: 10.3390/cells10112861 (PMC8616452; doi:10.3390/cells10112861)
Supplement: Supplementary file 1 [file cells-10-02861-s001.zip › cells-1406663-supplementary.pdf]

Supplementary material for:

'A-KINASE ANCHORING PROTEIN 2 PROMOTES PROTECTION AGAINST MYOCARDIAL INFARCTION'

Darko Maric[1,2], Aleksandra Paterek[1], Marion Delaunay[1], Irene Perez Lopez[1], Miroslav Arambasic[1], Dario Diviani[1*]

[1] *Department of Biomedical Sciences, Faculty of Biology and Medicine, University of Lausanne, 1011 Lausanne, Switzerland*

[2] *Current affiliation: Section of Medicine, Department of Endocrinology, Metabolism and Cardiovascular system, University of Fribourg, 1700 Fribourg, Switzerland*

[*] Corresponding author: Dario.diviani@unil.ch

This file includes:

Figures S1 to S5

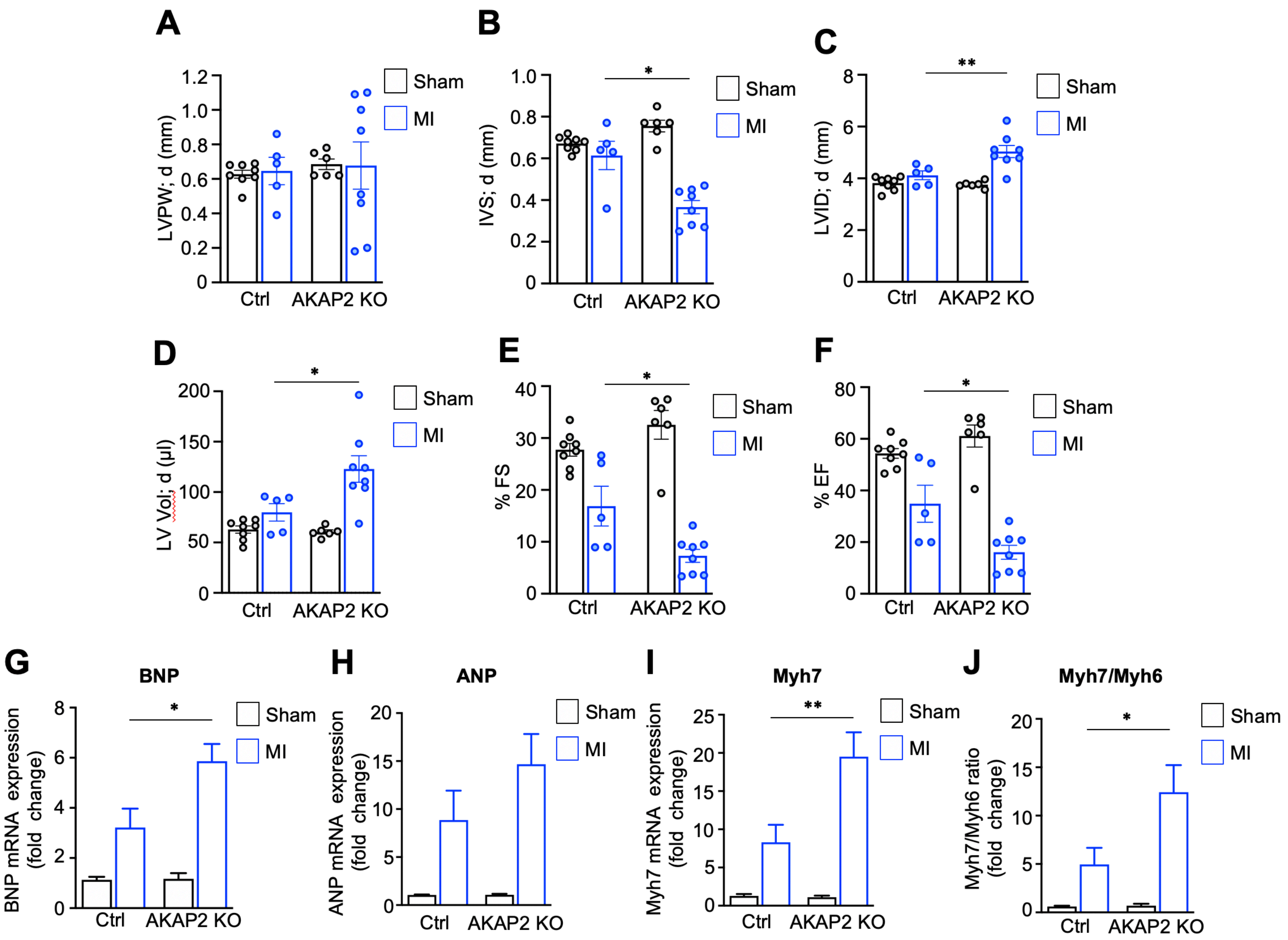

Figure S1. Targeted deletion of AKAP2 in adult cardiomyocytes exacerbates MI-induced cardiac dysfunction in female mice. A) End-diastolic left ventricular posterior wall thickness (LVPW; d). **B)** End-diastolic intraventricular septum thickness (IVS; d). **C)** End-diastolic left ventricular internal diameter (LVID; d). **D)** End-diastolic left ventricular volume (LV Vol; d). **E-F)** Fractional shortening (FS) and Ejection fraction (EF). Size of the experimental groups size for panels A-F: Ctrl-sham n=8, Ctrl-MI n=5, AKAP2 KO-sham n=6, AKAP2 KO-MI n=8. **G-J)** Quantitative real time PCR analysis of the expression of BNP (G), ANP (H), Myh7 (I). The Myh7:Myh6 ratio is shown (J). Size of the experimental groups size for panels G-J: Ctrl-sham n=4, Ctrl-MI n=4, AKAP2 KO-sham n=4, AKAP2 KO-MI n=4.Values are presented as mean ± SEM. *P < 0,05; **P < 0,01.

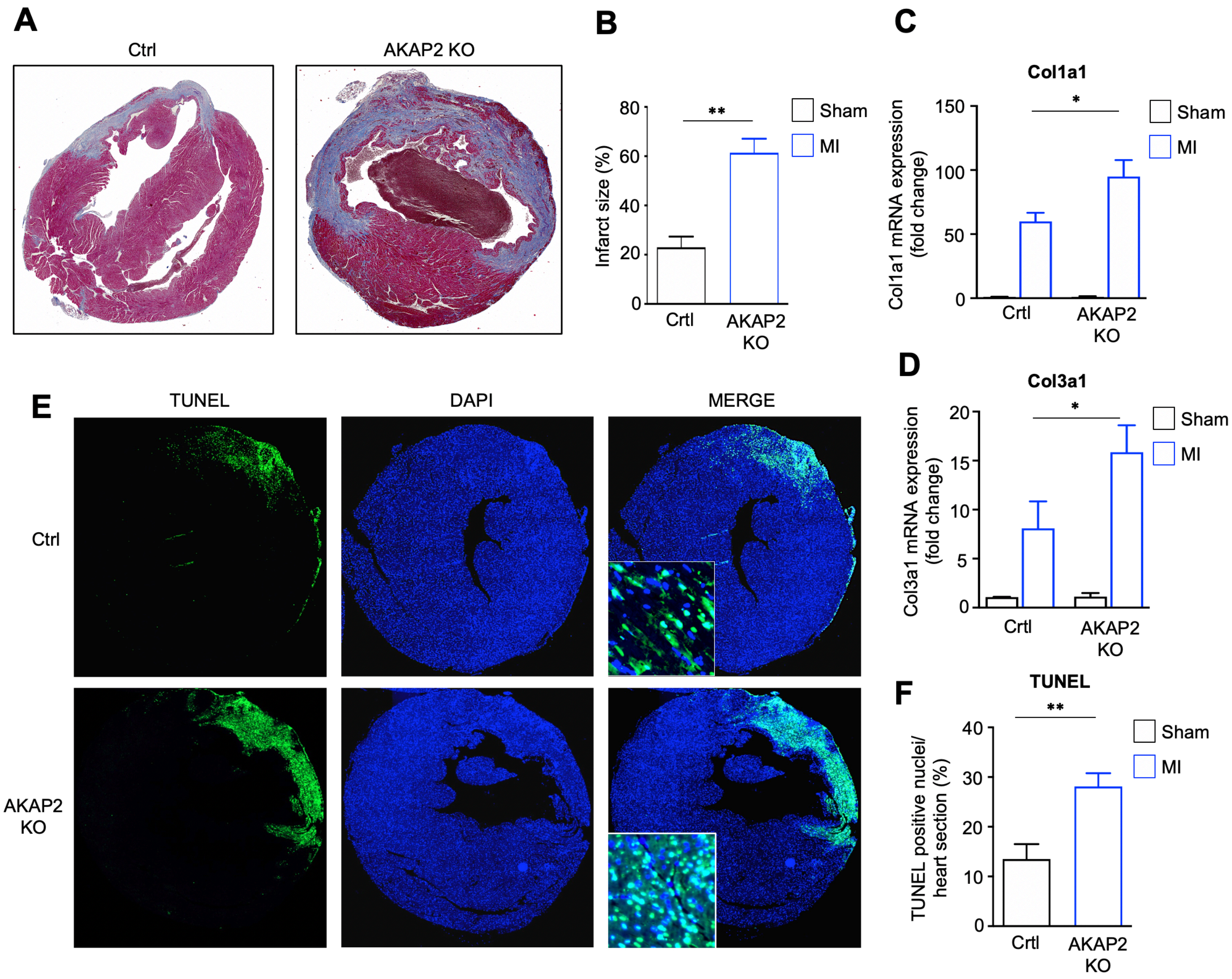

Figure S2. AKAP2 knockout in adult cardiomyocytes enhances myocardial apoptosis, fibrosis and infarct size in female mice after LAD ligation. A) Masson's Trichrome stainings performed on mid-ventricular heart sections from control (Ctrl) and cardiomyocyte-specific AKAP2 KO female mice 2 weeks after MI. **B)** Quantitative assessment of infarct size. For each heart, the proportion of scar tissue in the left ventricle was quantified on 3-4 mid-ventricular transversal sections stained with Masson's Trichrome. Ctrl-MI n=3 ; AKAP2 KO-MI n=5. **C-D)** The relative mRNA expression of collagen 1a1 (C) and 3a1 (D) in heart lysates from the indicated mice groups was assessed by quantitative real-time PCR. Values were normalized to the expression of GAPDH. Ctrl-MI n=4 ; Ctrl-sham n=4. **E)** Representative TUNEL and DAPI stainings of transversal heart sections taken from control (Ctrl) and cardiomyocyte-specific AKAP2 KO female mice 24 h after LAD ligation. **F)** Quantification of apoptosis. The number of TUNEL-positive nuclei was normalized to the total number of nuclei per heart section. Data from 5-6 different heart sections were averaged for each mouse. Ctrl-MI n=4 ; AKAP2 KO-MI n=5. Values are presented as mean ± SEM. *P < 0,05; **P < 0,01.

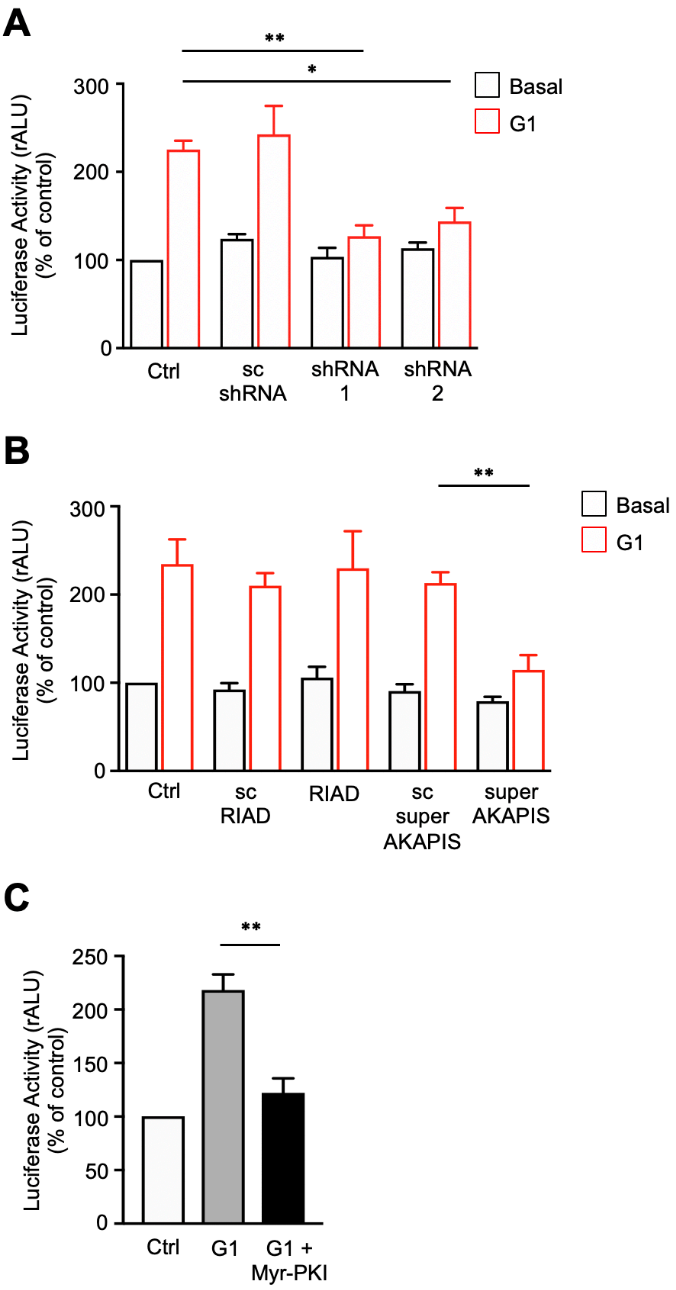

Figure S3. AKAP2 mediates GPR30-induded ER-activation in cardiomyocytes. A) Rat NVMs were infected using control lentiviruses or lentiviruses encoding wild type (AKAP2 shRNA 1 and 2) or scrambled (sc shRNA) AKAP2 shRNAs and subsequently transfected with ERE-firefly luciferase and renilla luciferase reporter constructs. 72 h after infection cells were serum-starved for 24 h and then incubated for 8 h in the absence or presence of 10^{-7} M G1. Firefly-luciferase activity was normalized to Renilla-luciferase activity. Results are the mean ± S.E of 4 independent experiments. **B)** Rat NVMs were transfected with ERE-firefly luciferase and renilla luciferase reporter constructs. 24h later and after a 24 h serum starvation cells were pre-treated for 45 min with 20 μM of scrambled RIAD, RIAD, scrambled super-AKAP-IS and super-AKAP-IS and subsequently incubated for 8 h with or without with 10^{-7} M G1. Firefly-luciferase activity was normalized to Renilla-luciferase activity. Results are the mean ± S.E of 3 independent experiments. **C)** Rat NVMs were transfected as indicated in B). 24h later cells were serum starved for 24 h and subsequently left untreated or incubated for 8 h with 10^{-7} M G1 in the presence or absence of 10^{-6} M Myr-PKI. Firefly-luciferase activity was normalized to Renilla-luciferase activity. Results are the mean ± S.E of 3 independent experiments. *P < 0,05; **P < 0,01.

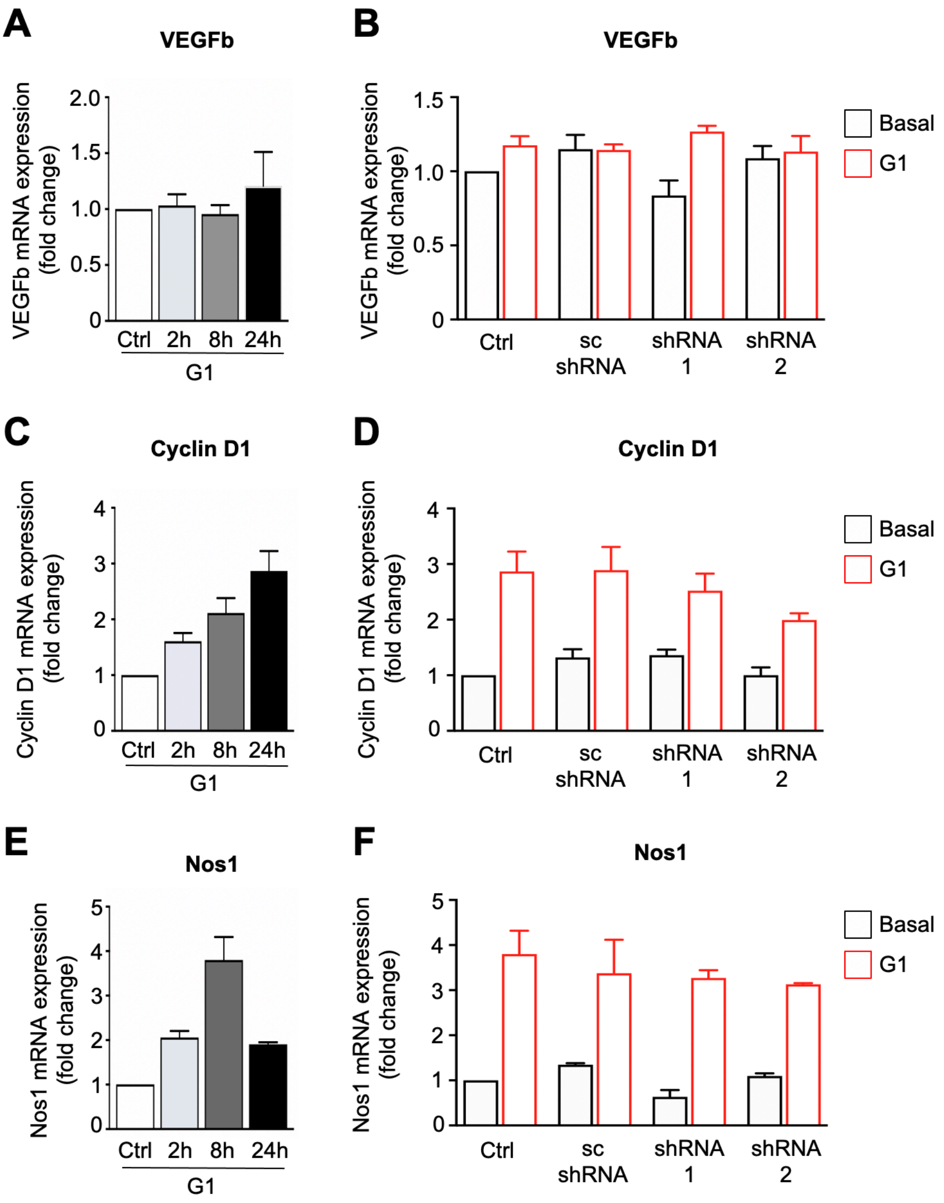

Figure S4. AKAP2 does not participate in the regulation of VEGFb, Cyclin D1 and Nos1. A, C, E) Time course of G-1-induced VEGFb (A), Cyclin D1 (C), and Nos1 (E) mRNA expression. Rat NVMs were serum starved for 24 h and subsequently treated with 10^{-7} M G1 for the indicated periods of time. VEGFb, Cyclin D1, and Nos1 mRNA expression was assessed by quantitative real-time PCR. **B, D, F)** Rat NVMs were infected using control (Ctrl) lentiviruses or lentiviruses encoding wild type (shRNA 1 and 2) or scrambled (sc) AKAP2 shRNAs. 72 h after infection cells were serum starved for 24 h and subsequently treated with 10^{-7} M G1 for 24h. VEGFb, Cyclin D1, and Nos1 mRNA expression was determined by quantitative PCR. Results are expressed as mean ± S.E. of 3 experiments.

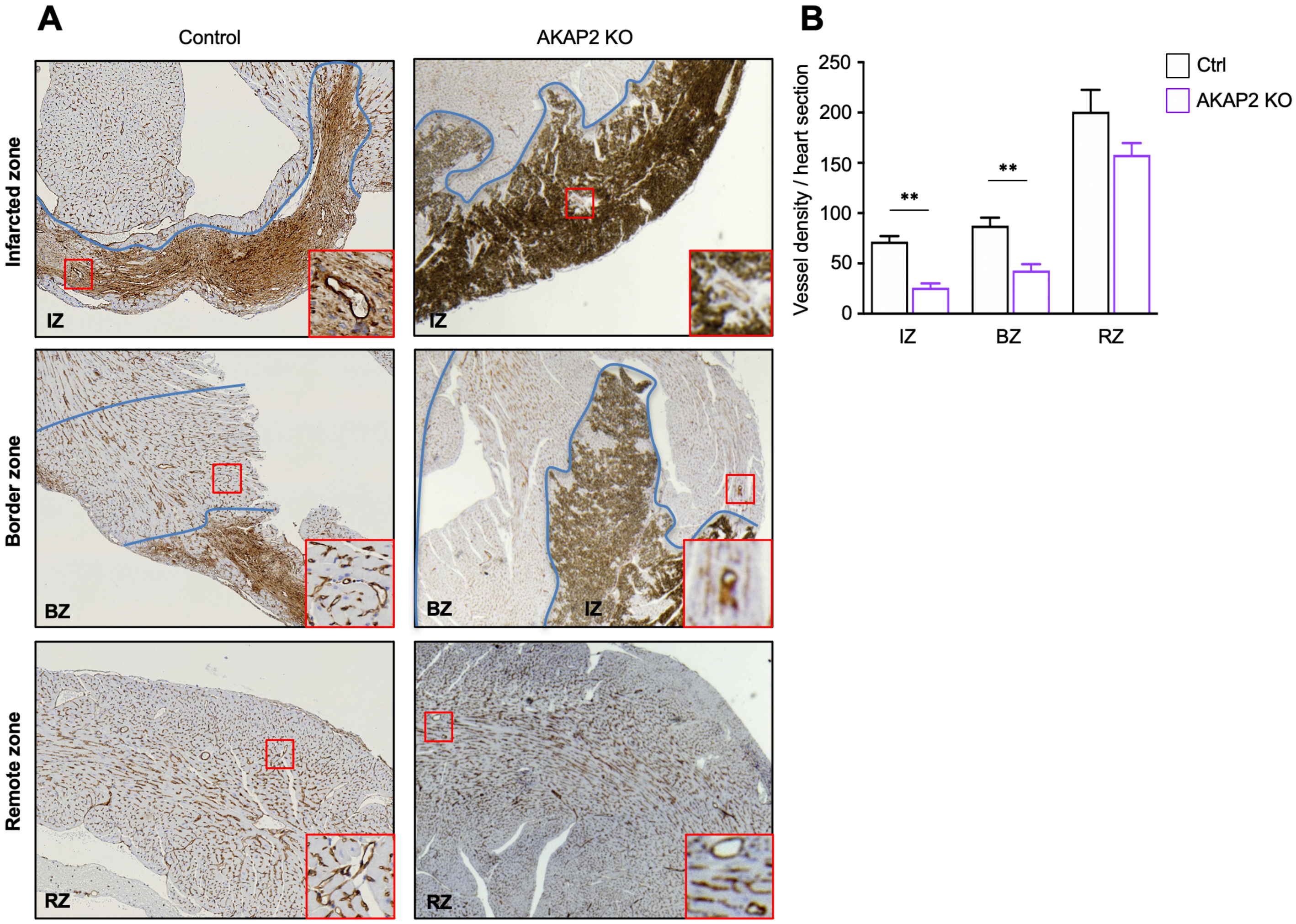

Figure S5. AKAP2 knockout in adult cardiomyocytes reduces vessel number in infarcted and border zones of female mice. A) Control (Ctrl) or cardiomyocyte-specific AKAP2 KO female mice underwent sham operation or LAD ligation. 2 weeks after surgery, hearts were harvested and included in paraffin. Deparaffinized-heart sections were stained for lectin. **B)** For each heart, the number of vessels was quantified on 3-4 heart sections using the ImageJ software. Vessel density was reported for infarcted (IZ), border (BZ) and remote zones (RZ). Results are presented as the mean ± SEM. Ctrl-MI n=3, AKAP2 KO-MI n=4. **P < 0,01.
